# Supplementary material for: scGCN is a graph convolutional networks algorithm for knowledge transfer in single cell omics
Source: Nat Commun. 2021 Jun 22;12:3826. doi: 10.1038/s41467-021-24172-y (PMC8219725; doi:10.1038/s41467-021-24172-y)
Supplement: Supplementary file 1 — Supplementary Information [file 41467_2021_24172_MOESM1_ESM.pdf]

SUPPLEMENTARY INFORMATION

scGCN is a Graph Convolutional Networks Algorithm for Knowledge Transfer in Single Cell Omics

Supplementary Figures ---------------- 3-18

Supplementary Notes ---------------- 19-30

Supplementary References ---------------- 31

Codes functionality ---------------- 32-33

Supplementary Fig. 1 Performance of CHETAH and scmap.. 3
Supplementary Fig. 2 Performance of Conos on A549 dataset. 4
Supplementary Fig. 3 Application of scGCN to mouse brain dataset. 5
Supplementary Fig. 4. Batch effects evaluation of CCA and scGCN. 6
Supplementary Fig. 5 Label transfer accuracy of CCA-MNN and scGCN. 7
Supplementary Fig. 6 Performance of scGCN on original and reversed reference-query data pairs. 8
Supplementary Fig. 7 Performance of scGCN on comprehensive benchmarking datasets... 9
Supplementary Fig. 8 Computational performance of scGCN on large-size single-cell dataset. 10
Supplementary Fig. 9 Performance of overall-learning versus batch-splitting on large-size single-cell dataset. 11
Supplementary Fig. 10 Accuracy of graphs constructed by different methods. 13
Supplementary Fig. 11 Performance of GCN based on different constructed graphs. 16
Supplementary Fig. 12 Robustness of GCN postprocessing based on CCA-MNN constructed graphs. 16
Supplementary Fig. 13 Performance of label transfer using different graph neural networks. 17
Supplementary Fig. 14 Performance of entropy score and enrichment score in identifying unknow cells in query data 18
Supplementary Fig. 15 Performance of CCA-based Seurat v3, PCA-based Seurat v3, and scGCN. 19
Note 1: Comparisons of scGCN with CCA for overcoming batch effects 20
Note 2: Performance evaluation of scGCN using additional benchmarking data 21
Note 3: Computational scalability of scGCN on large-size single-cell data 22
Note 4: Effectiveness of the hybrid graph in scGCN 24
Note 5: Comparisons of GCN model with other graph neural network models 27
Note 6: Evaluate the capability of scGCN for detecting unknown cells 29
Note 7: Compare scGCN with CCA-based Seurat v3 in label transfer 31
References 32
Codes functionality 33

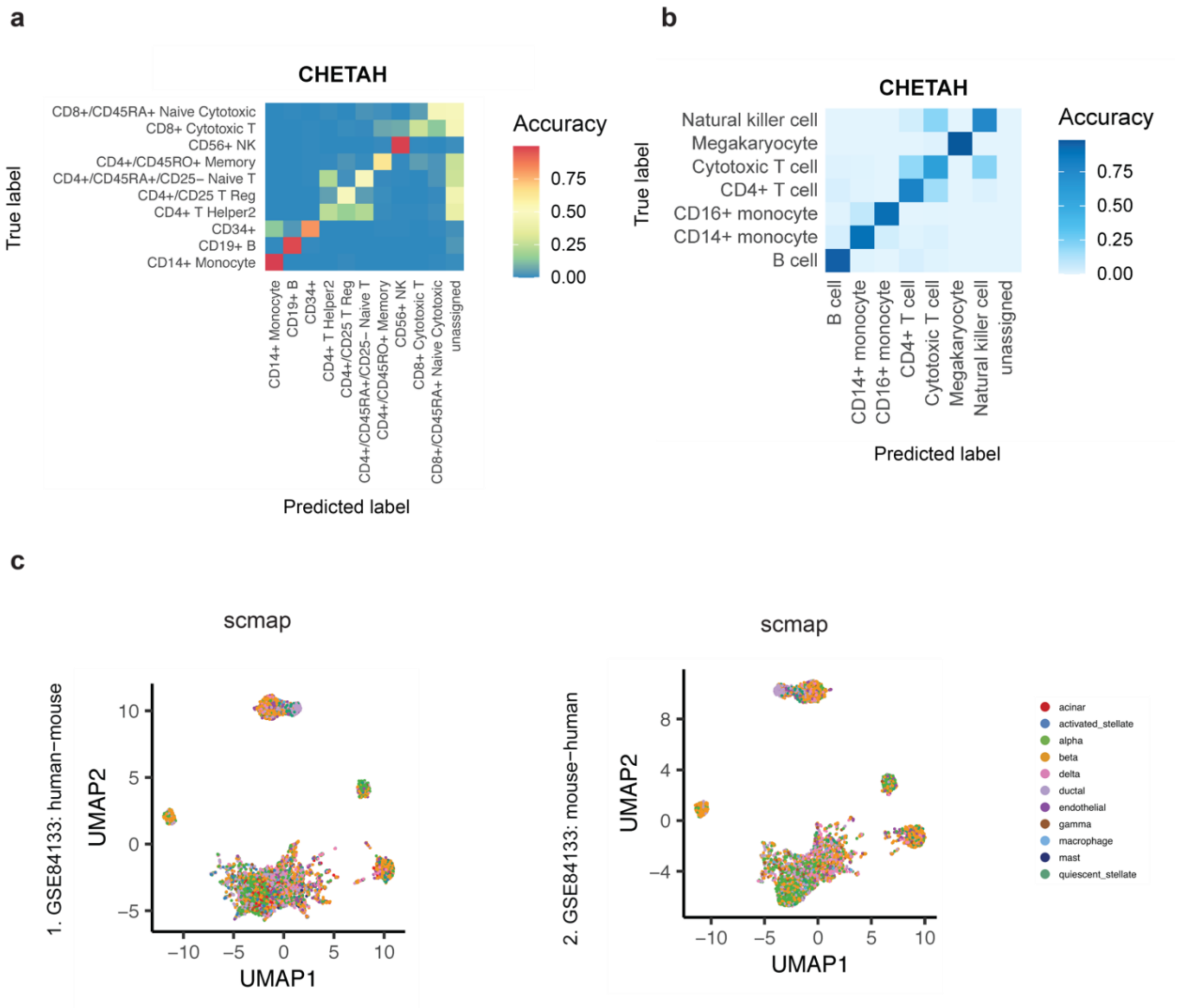

Supplementary Fig. 1 Performance of CHETAH and scmap. a Heatmap of the accuracy matrix of each cell type identified by CHETAH based on the single cell dataset (SRP073767). **b** Heatmap shows the accuracy matrix of each cell type identified by CHETAH based on the Cel-seq data as reference and the 10x V2 data as query set. **c** UMAP projection of the aggregated data of GSE84133 by scmap. The first UMAP represents the aggregated data using the human data as the reference and mouse data as the query data. The second row uses the mouse data as reference and human data as query.

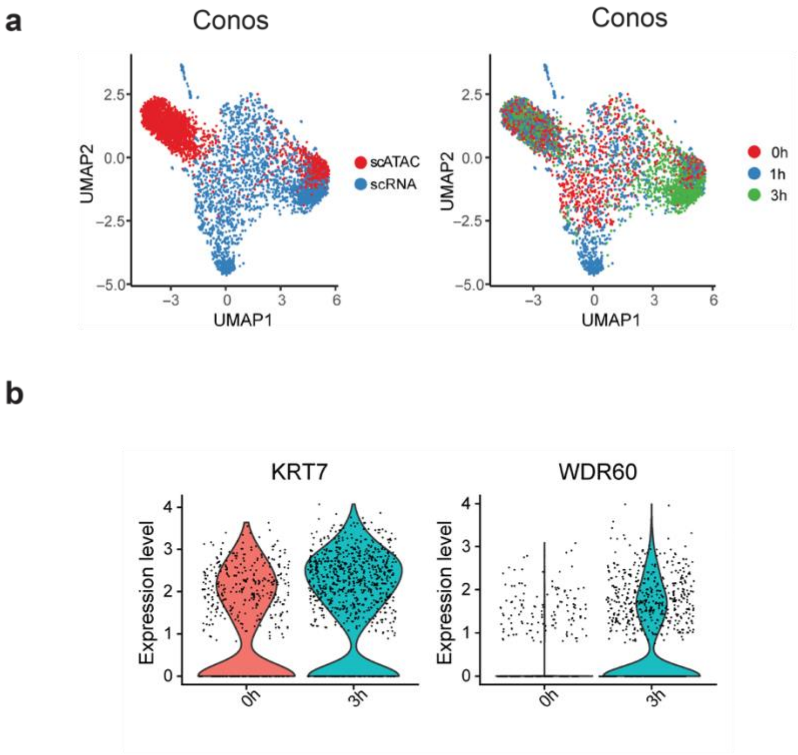

Supplementary Fig. 2 Performance of Conos on A549 dataset. a UMAP plots of A549 cells colored by dataset (scRNAseq, scATAC-seq) and by cell states (0h, 1h, 3h), after integration by Conos. **b** KRT7 and WDR60 are shown as overrepresented DNA motifs that are identified in 3h-specific accessibility peaks, which also exhibit overexpression in 3h cells.

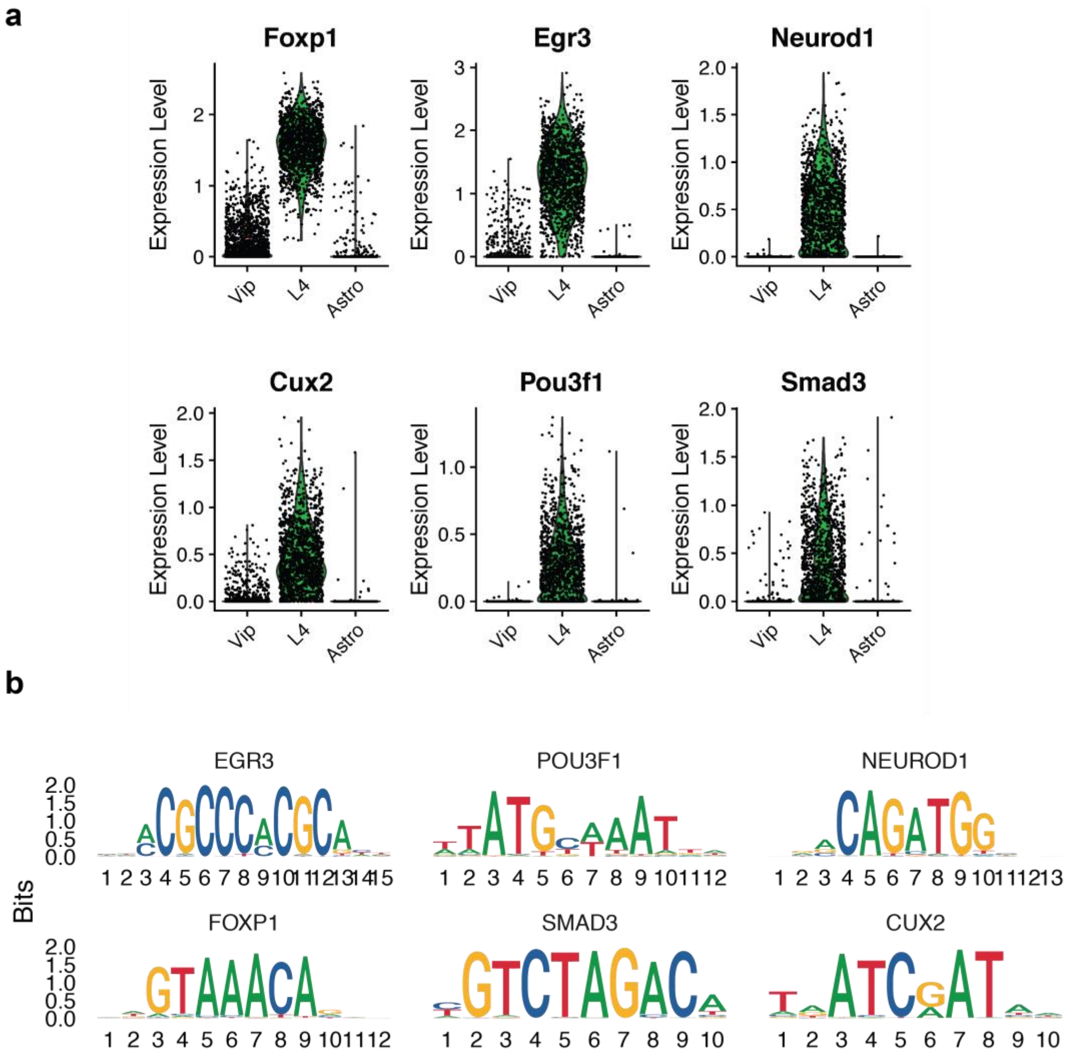

Supplementary Fig. 3 Application of scGCN to mouse brain dataset. a Overrepresented DNA motifs are identified in L4-specific accessibility peaks, with Foxp1, Egr3, and Smad3 motifs as the most highly enriched motifs. **b** These motifs also exhibit upregulated expression in L4 cell subtype.

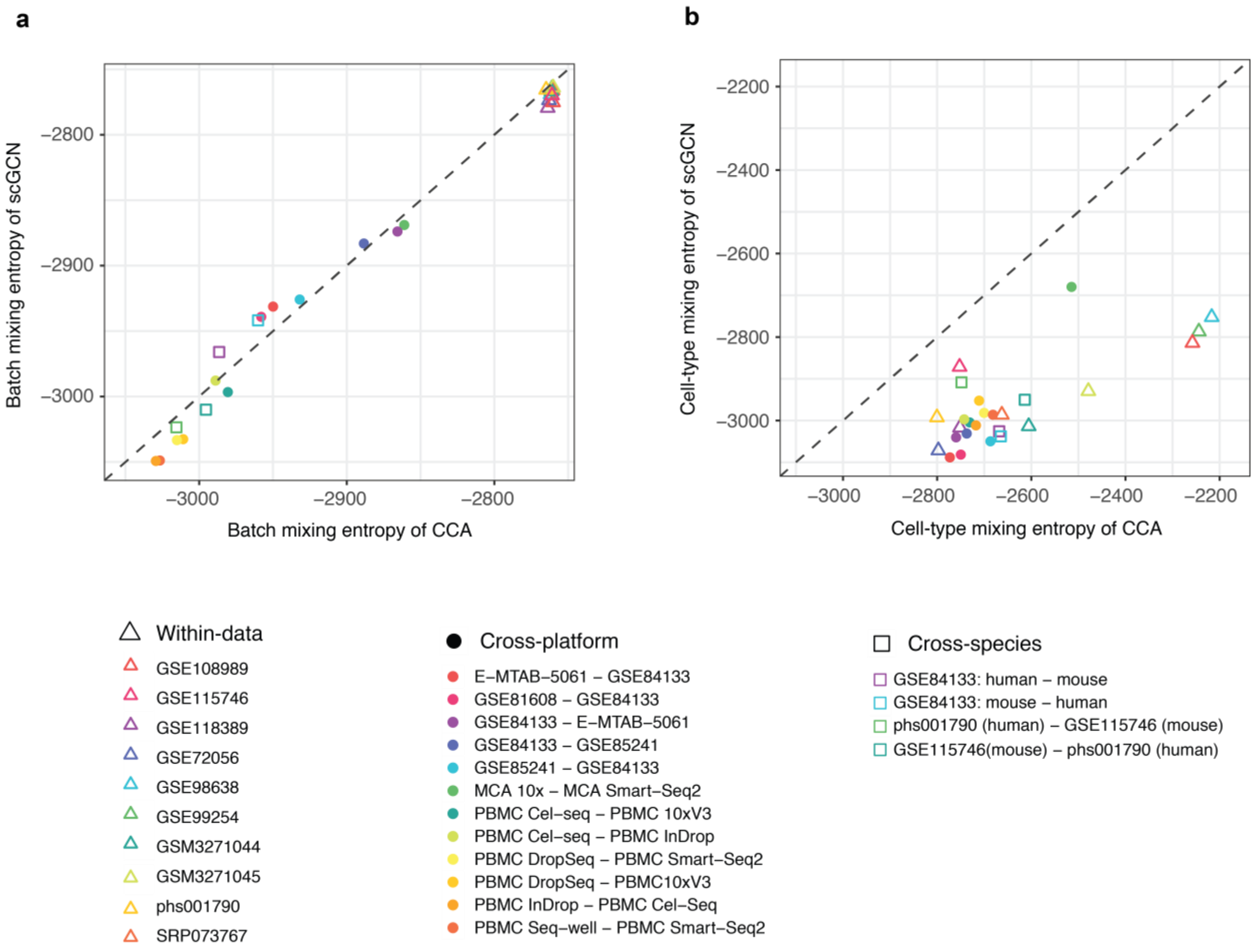

Supplementary Fig. 4. Batch effects evaluation of CCA and scGCN. CCA and scGCN are evaluated by the batch mixing entropy **(a)** and the cell-type mixing entropy **(b)** respectively. Different colors represent different paired reference-query datasets, while different symbols represent different label transfer scenarios, i.e., within-data, cross-platform, cross-species. The dashed line represents equivalent mixing entropy between two methods. Source data are provided as a Source Data file.

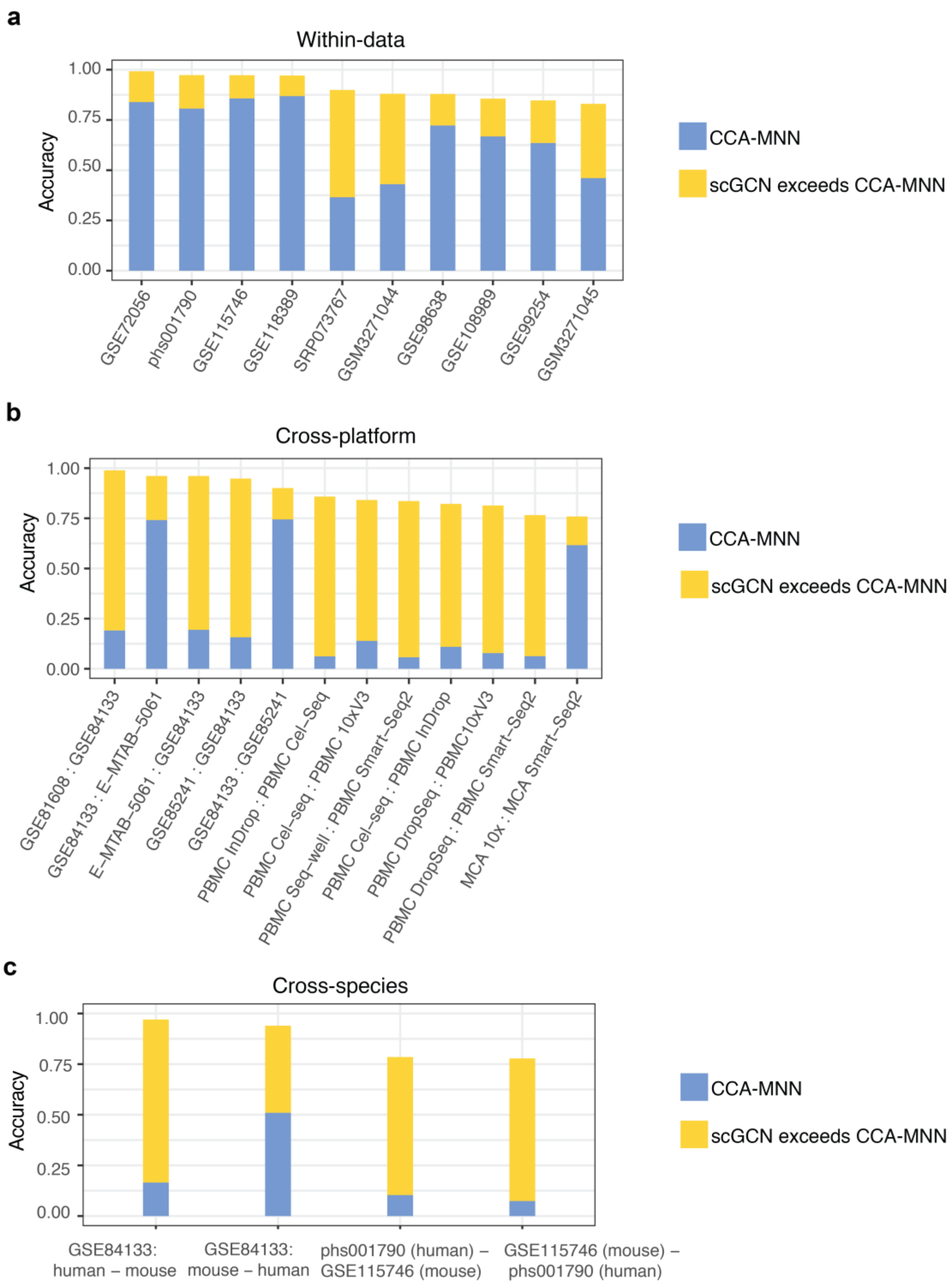

Supplementary Fig. 5 Label transfer accuracy of CCA-MNN and scGCN. Three panels **a - c** show the accuracy of labels identified by CCA-MNN and scGCN, based on the reference-query pairs from within-data **(a)**, cross-platform **(b)**, and cross-species datasets **(c)**, respectively. The blue-colored bar represents the accuracy of CCA-MNN, while the yellow-colored bar represents

the improved accuracy from the post-processing GCN model. Source data are provided as a Source Data file.

a

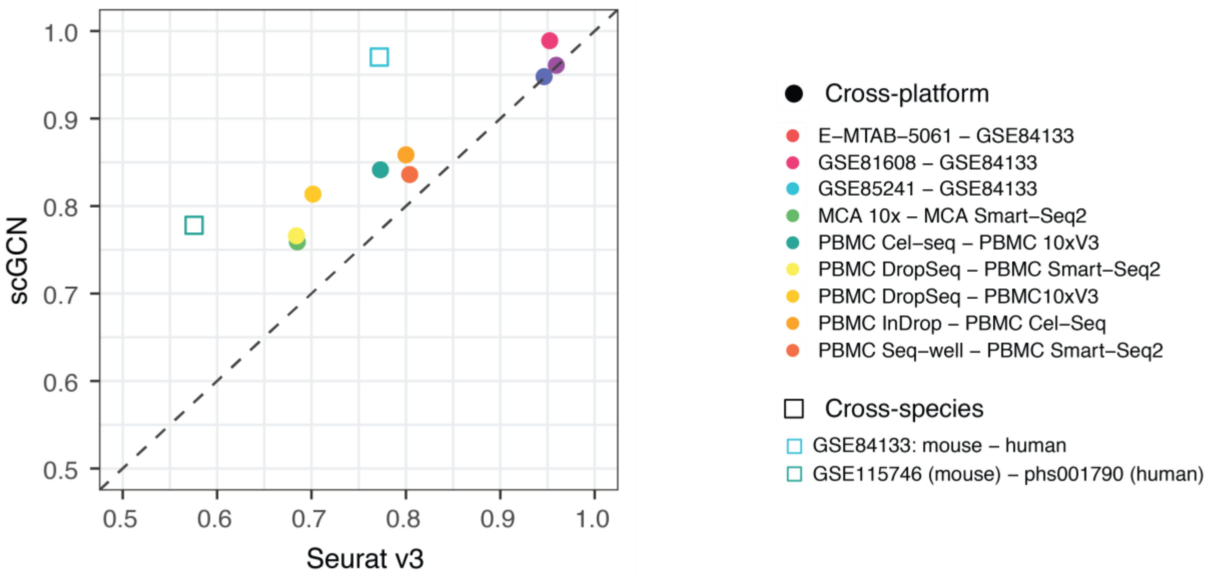

b

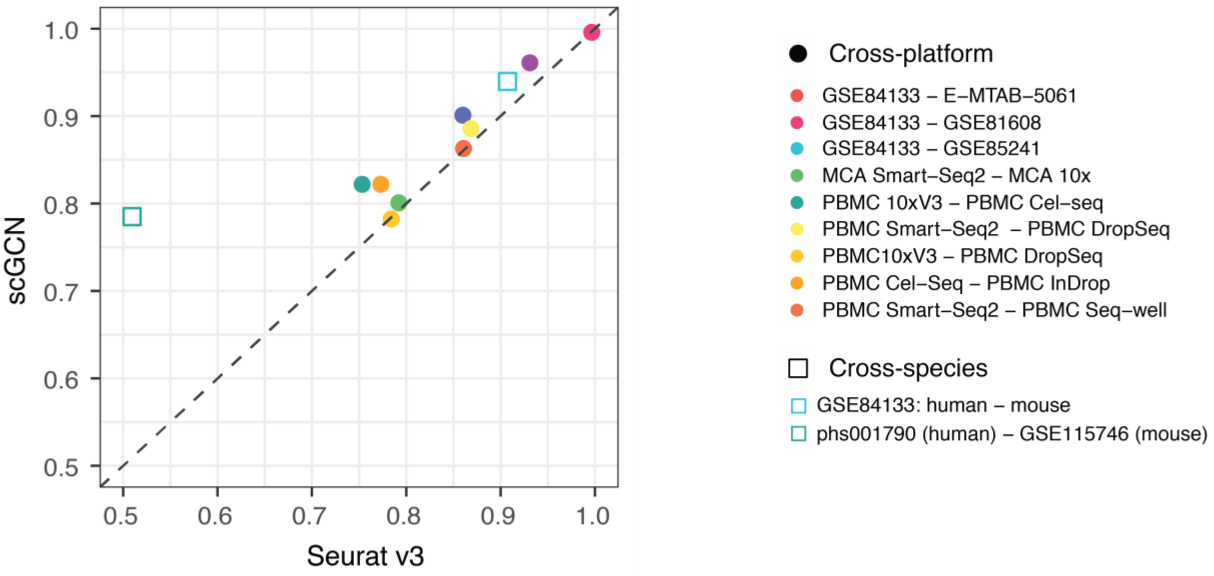

Supplementary Fig. 6 Performance of scGCN on original and reversed reference-query data pairs. a Performance of scGCN versus Seurat v3 on the 11 unique pairs of reference-query data from Fig. 3. **b** Performance of scGCN versus Seurat v3 on the 11 reversed pairs of reference-query data. The dashed line represents equivalent accuracy between two methods. Different colored symbols represent different reference-query pairs. Source data are provided as a Source Data file.

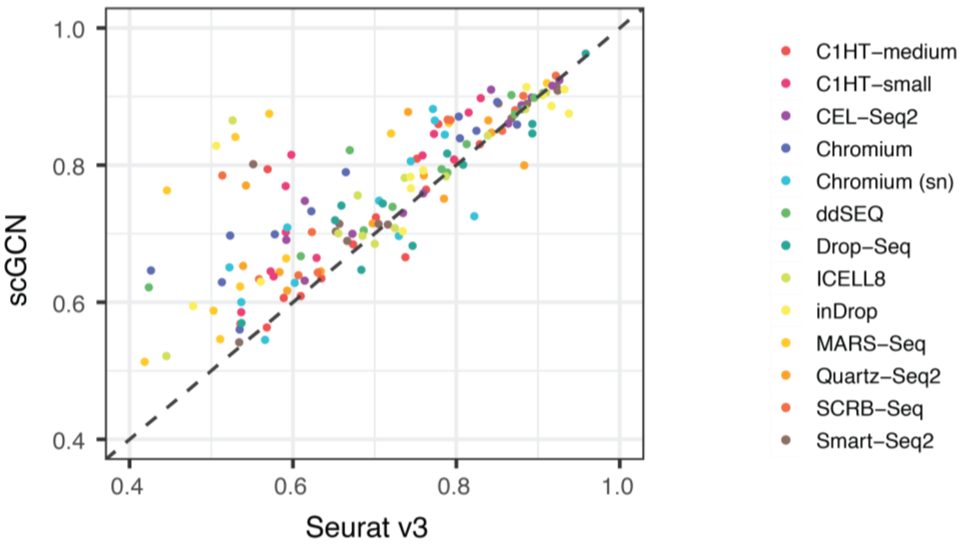

Supplementary Fig. 7 Performance of scGCN on comprehensive benchmarking datasets. Performance of scGCN versus Seurat v3 on 156 reference-query pairs from 13 different platforms, including C1HT-medium, MARS-Seq, Quartz-Seq2, SCRB-Seq, Smart-Seq2, C1HT-small, CEL-Seq2, Chromium, Chromium (sn), ddSEQ, Drop-Seq, ICELL8, and inDrop. The dashed line represents equivalent accuracy between two methods. Different colors represent reference-query pairs from different platforms. Source data are provided as a Source Data file.

a

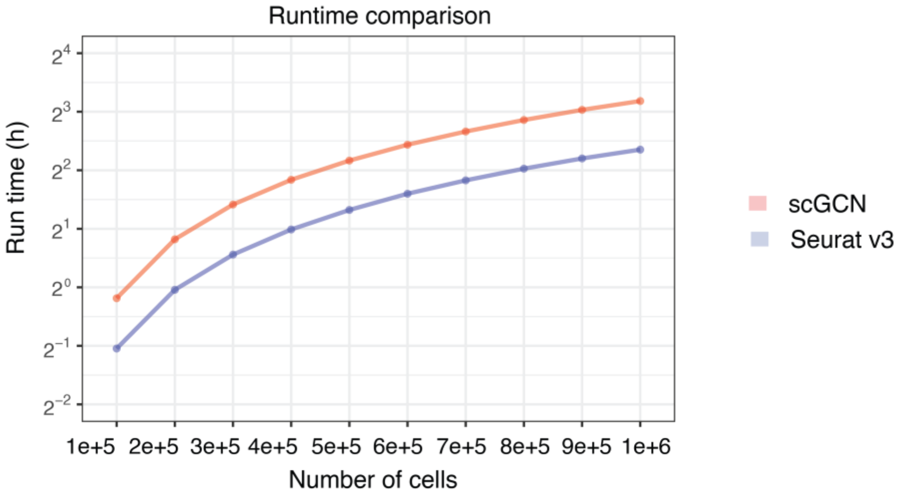

b

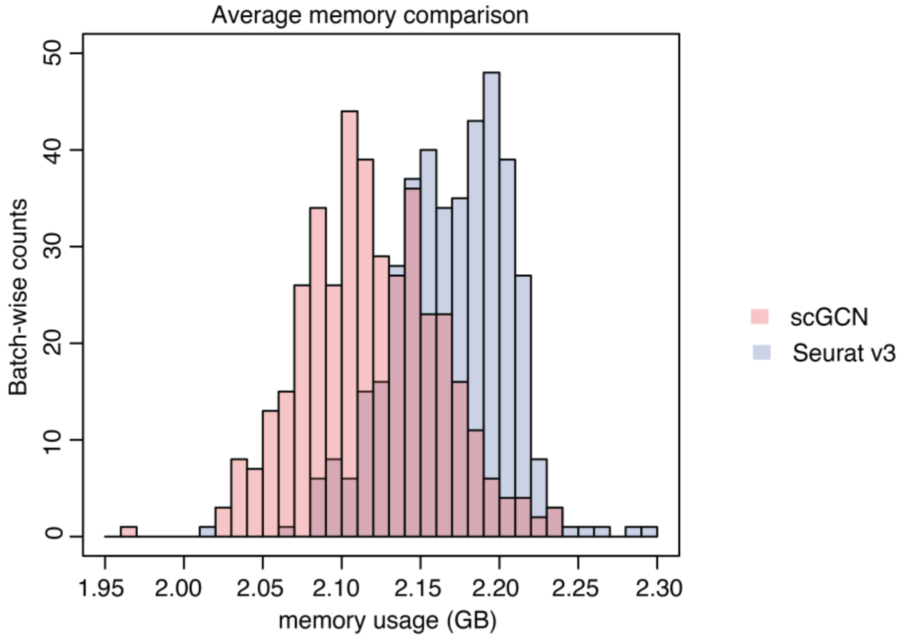

Supplementary Fig. 8 Computational performance of scGCN on large-size single-cell dataset. **a** Computation time of scGCN and Seurat on datasets of different sample sizes. The x-axis represents different sample sizes, the y-axis represents the corresponding running time in hours. **b** Batch-wise average memory usage of scGCN and Seurat across batches. The x-axis represents the memory usage (GB) and the y-axis represents the counts of batches, with each batch consisting of 5, 000 cells. Source data are provided as a Source Data file.

a

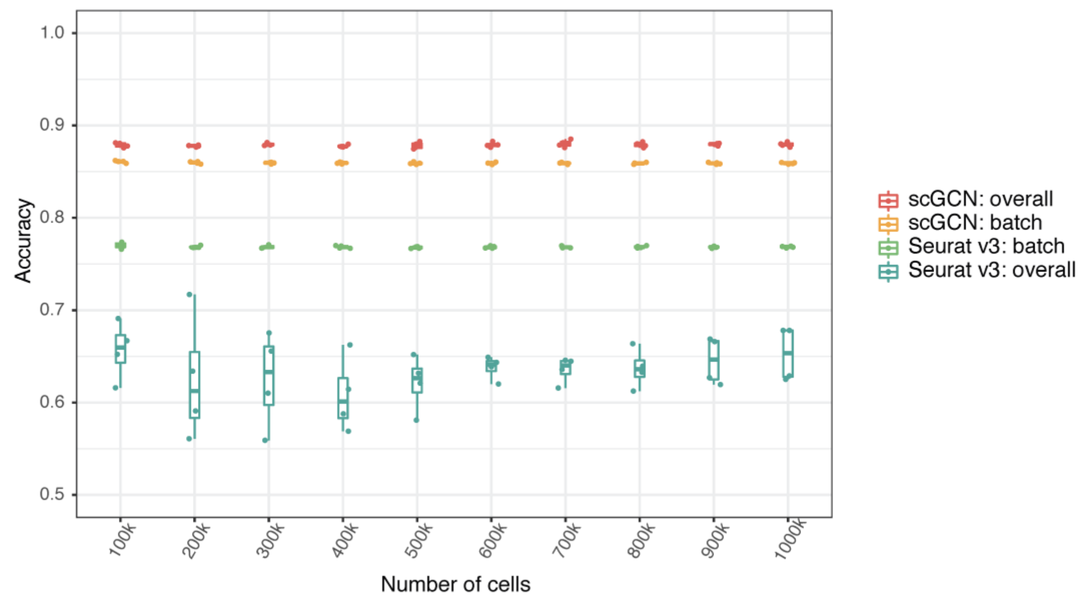

b

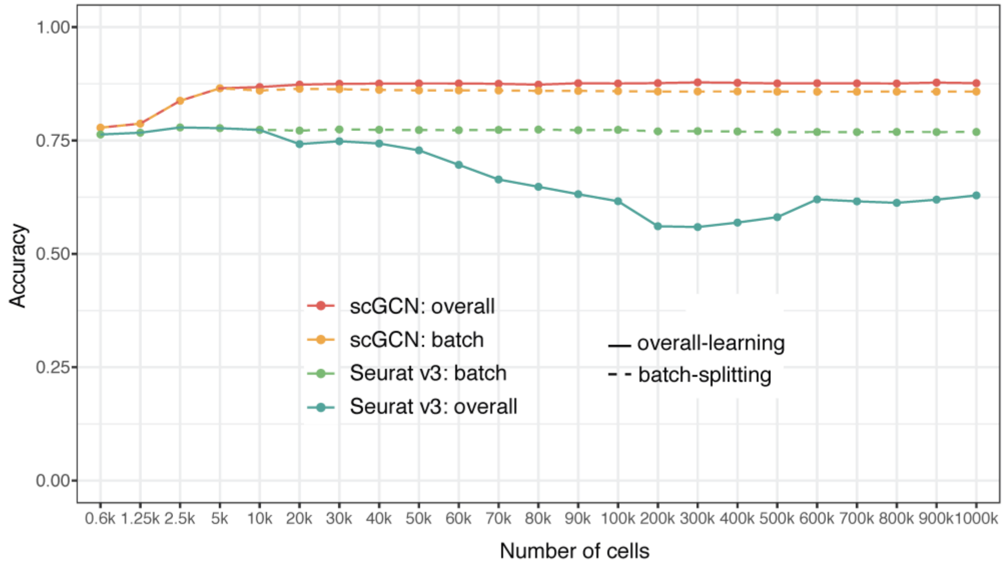

Supplementary Fig. 9 Performance of overall-learning versus batch-splitting on large-size single-cell dataset. a Accuracy of scGCN and Seurat v3 on 4 randomly sampled datasets of different cell numbers using batch-splitting and overall-learning, respectively. Data are represented as boxplots where the middle line is the median, the lower and upper hinges correspond to the first and third quartiles, the upper whisker extends from the hinge to the largest value no further than 1.5 × IQR from the hinge (where IQR is the inter-quartile range) and the lower whisker

extends from the hinge to the smallest value at most 1.5 × IQR of the hinge. **b** Accuracy of scGCN and Seurat v3 on sample sizes ranging from 0.6k to 1 million. X-axis represents cases of different samples sizes. Solid lines represent overall-learning results and dashed lines represent batch-splitting results. Different colors represent different methods. Source data are provided as a Source Data file.

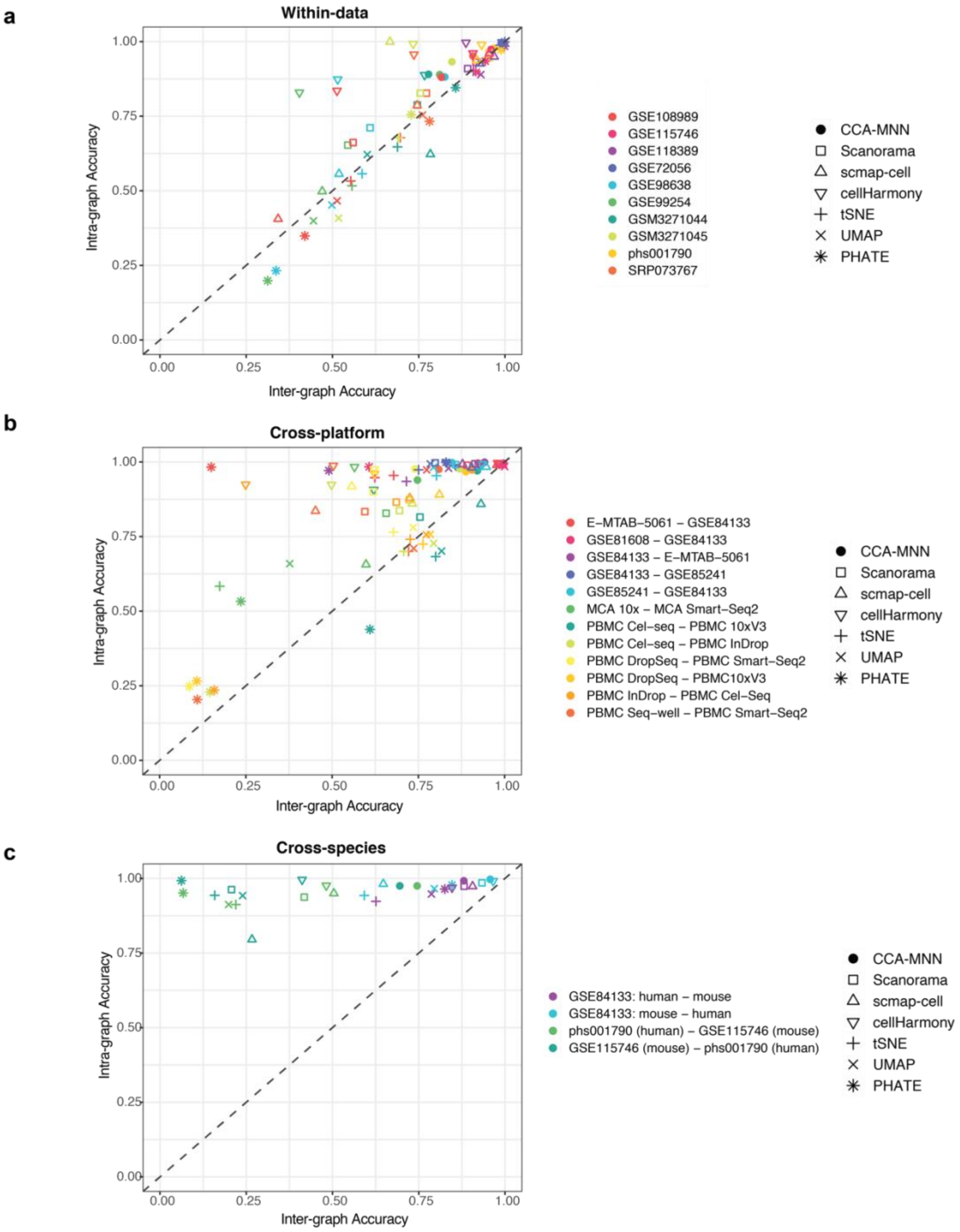

Supplementary Fig. 10 Accuracy of graphs constructed by different methods. CCA-MNN and other graph construction methods (Scanorama, scmap-cell, cellHarmony, tSNE, UMAP, PHATE) are evaluated by the intra-graph accuracy and inter-graph accuracy in the within-data **(a)**, cross-platform **(b)**, and cross-species scenarios **(c)**. Each symbol represents the inter-graph accuracy (x-axis) and the corresponding intra-graph accuracy (y-axis) of each method on a certain dataset. Different shapes indicate different methods while different colors represent different datasets. The

dashed line shows equivalent inter-graph accuracy and intra-graph accuracy. Source data are provided as a Source Data file.

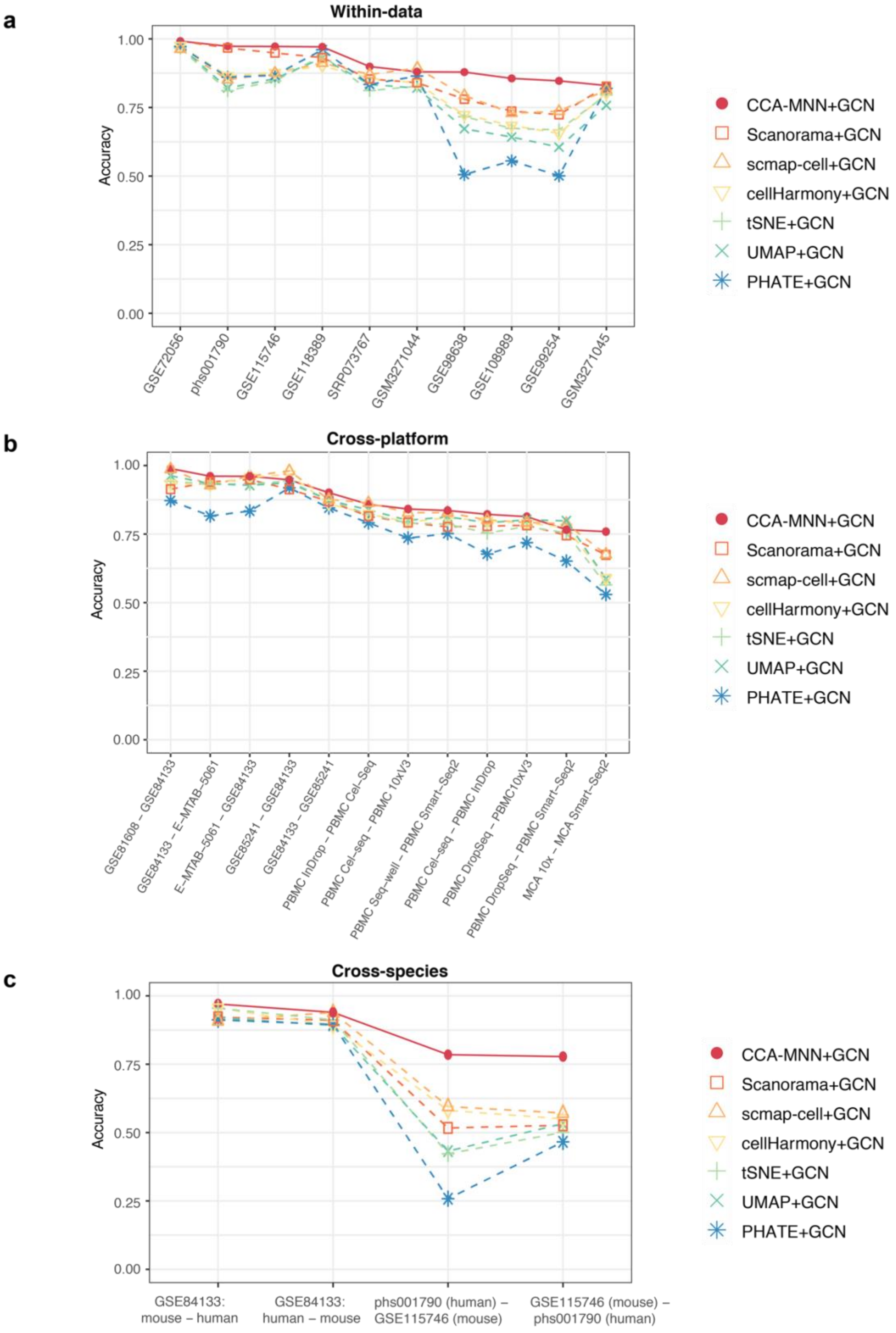
a
Within-data
Accuracy
1.00
0.75
0.50
0.25
0.00
GSE72056
phs001790
GSE115746
GSE118389
SRP073767
GSM3271044
GSE98638
GSE108989
GSE99254
GSM3271045
CCA-MNN+GCN
Scanorama+GCN
scmap-cell+GCN
cellHarmony+GCN
tSNE+GCN
UMAP+GCN
PHATE+GCN
b
Cross-platform
Accuracy
GSE81608 – GSE84133
GSE84133 – E-MTAB-5061
E-MTAB-5061 – GSE84133
GSE85241 – GSE84133
GSE84133 – GSE85241
PBMC InDrop – PBMC Cel-Seq
PBMC Cel-seq – PBMC 10xV3
PBMC Seq-well – PBMC Smart-Seq2
PBMC Cel-seq – PBMC InDrop
PBMC DropSeq – PBMC10xV3
PBMC DropSeq – PBMC Smart-Seq2
MCA 10x – MCA Smart-Seq2
c
Cross-species
Accuracy
GSE84133: mouse – human
GSE84133: human – mouse
phs001790 (human) – GSE115746 (mouse)
GSE115746 (mouse) – phs001790 (human)

Supplementary Fig. 11 Performance of GCN based on different constructed graphs. Performance of GCN using different graph construction methods, i.e., CCA-MNN+GCN, Scanorama+GCN, scmap-cell+GCN, cellHarmony+GCN, tSNE+GCN, UMAP+GCN, and PHATE+GCN, is measured by the accuracy score in the in the within-data **(a)**, cross-platform **(b)**, and cross-species **(c)** scenarios. Each shape with corresponding color represents each of the different methods. Solid line is shown above the dashed lines demonstrating that scGCN outperforms the other methods across these datasets. Source data are provided as a Source Data file.

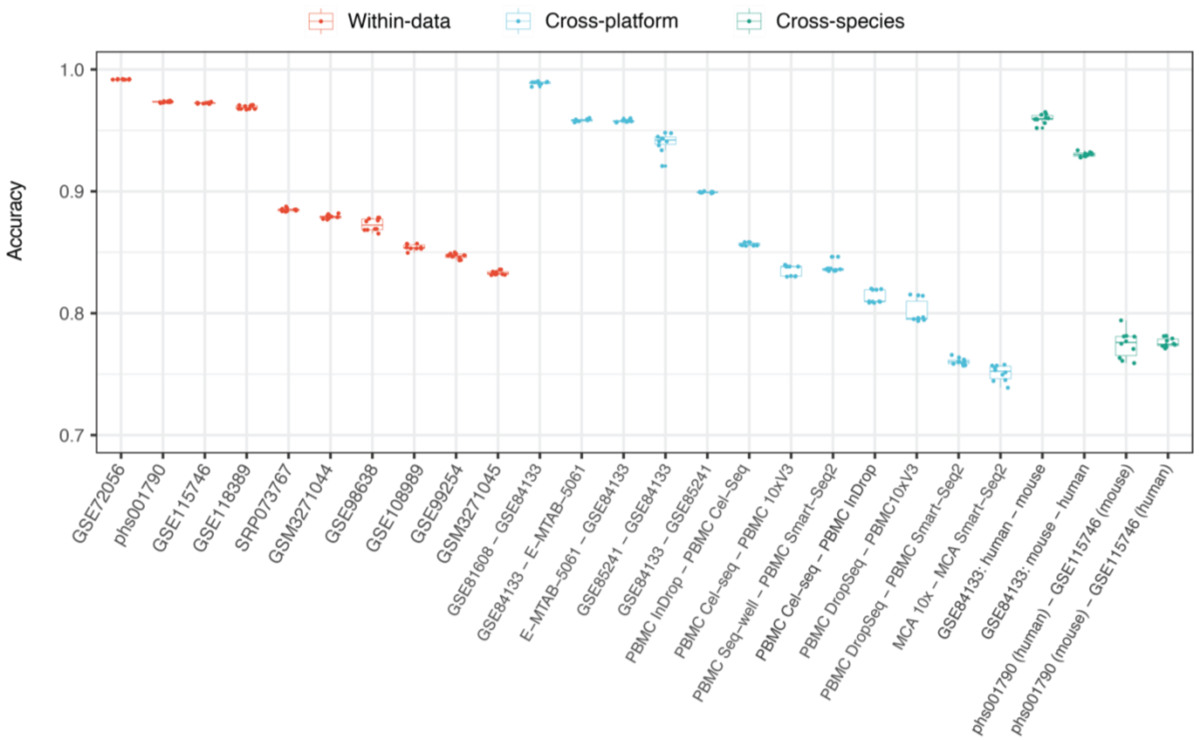

Supplementary Fig. 12 Robustness of GCN postprocessing based on CCA-MNN constructed graphs. For each reference-query pair from different scenarios, i.e., within-data, cross-platform, and cross-species, we use 10 random initializations of scGCN model and examine the accuracy scores accordingly. Data are represented as boxplots where the middle line is the median, the lower and upper hinges correspond to the first and third quartiles, the upper whisker extends from the hinge to the largest value no further than 1.5 × IQR from the hinge (where IQR is the inter-quartile range) and the lower whisker extends from the hinge to the smallest value at most 1.5 × IQR of the hinge. Source data are provided as a Source Data file.

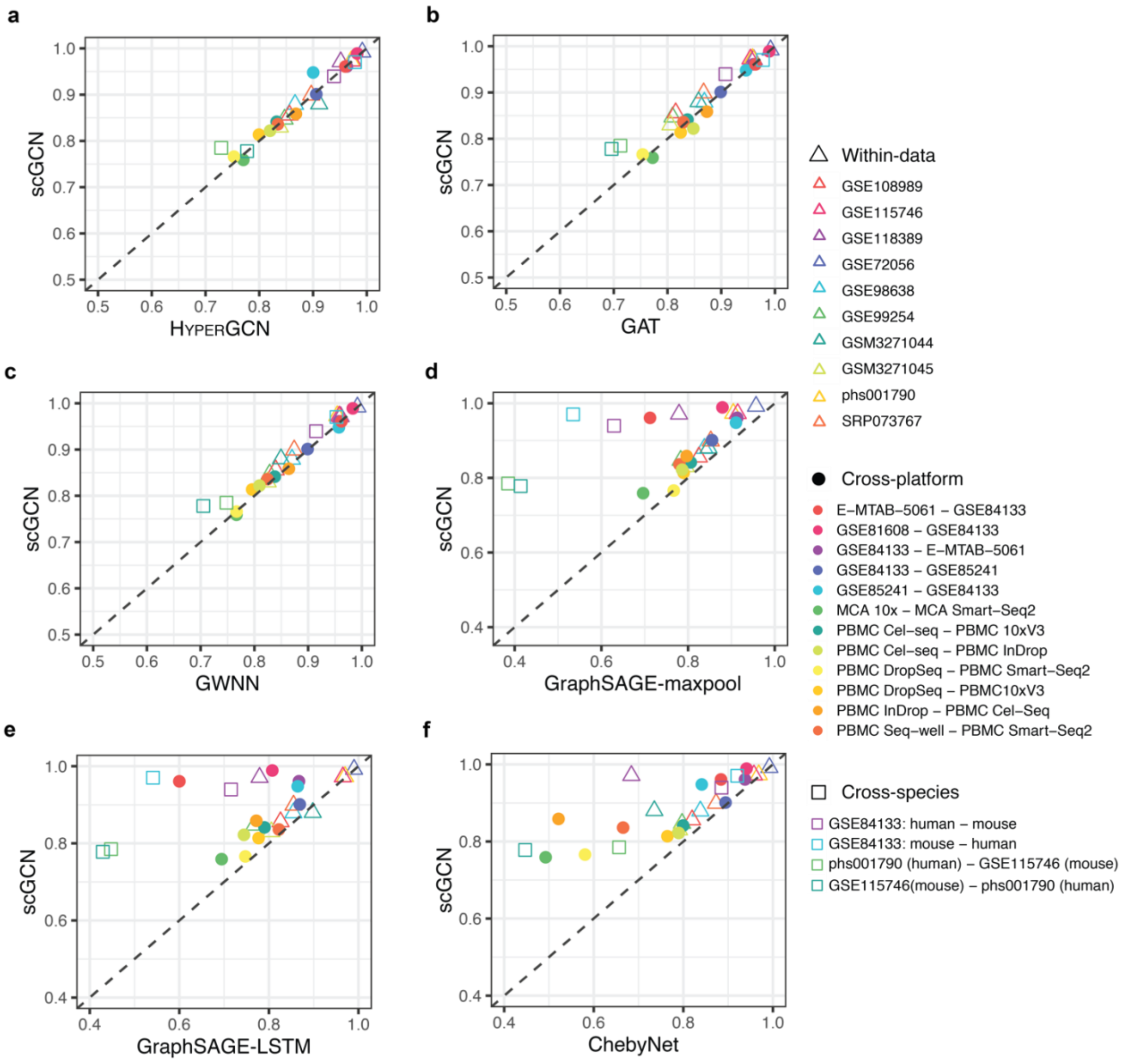

Supplementary Fig. 13 Performance of label transfer using different graph neural networks. Performance of scGCN and other methods including HYPERGCN **(a)**, GAT **(b)**, GWNN **(c)**, GraphSAGE-maxpool **(d)**, GraphSAGE-LSTM **(e)**, and ChebyNet **(f)** is measured by the accuracy score on all 26 datasets. Different colors represent different paired reference-query datasets, while different symbols represent different label transfer scenarios, i.e., within-data, cross-platform, cross-species. The dashed line represents equivalent accuracy between two methods. Source data are provided as a Source Data file.

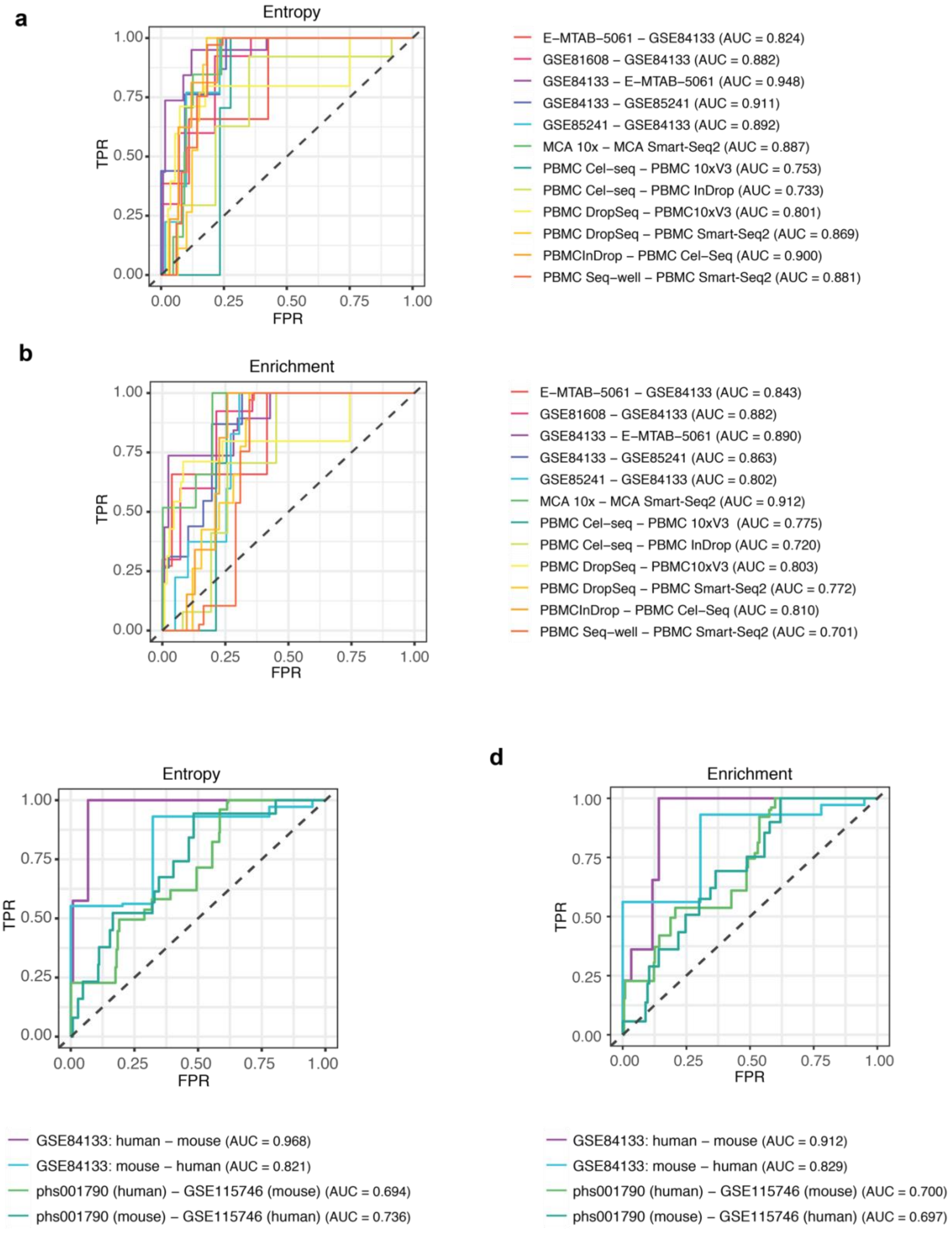

Supplementary Fig. 14 Performance of entropy score and enrichment score in identifying unknow cells in query data. ROC curves of entropy score **(a)** and enrichment score **(b)** on the 12 pairs of reference-query data from different platforms. ROC curves of entropy score **(c)** and

enrichment score **(d)** on the 4 pairs of reference-query data from different species. Different colors represent different reference-query pairs. The corresponding AUC score is indicated within the parenthesis. Source data are provided as a Source Data file.

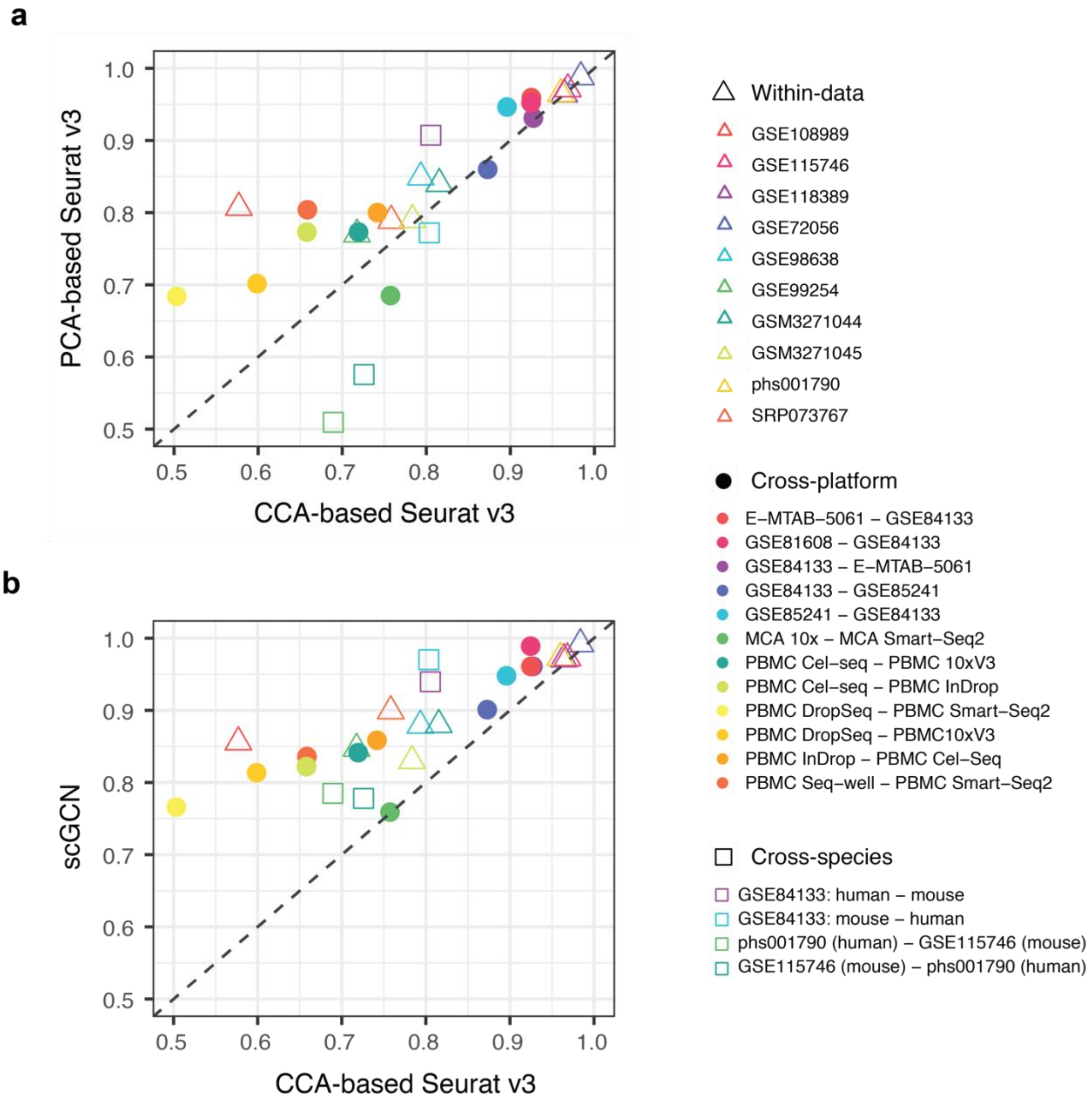

Supplementary Fig. 15 Performance of CCA-based Seurat v3, PCA-based Seurat v3, and scGCN. a Performance of CCA-based Seurat v3 versus PCA-based Seurat v3. **b** Performance of CCA-based Seurat v3 versus scGCN. The dashed line represents equivalent accuracy between two methods. Different symbols represent different learning scenarios, i.e., within-data, cross-platform, and cross-species. Different colors represent different paired reference-query datasets. Source data are provided as a Source Data file.

SUPPLEMENTARY NOTES

Note 1: Comparisons of scGCN with CCA for overcoming batch effects

To illustrate the contributions of scGCN for overcoming batch effects, we provide comparisons in two aspects. First, we evaluate the capability of CCA and scGCN in reducing batch effects while preserving cell type differences between datasets. Second, we evaluate the ability of CCA and scGCN for overcoming batch effects by their label transfer performance.

1). Reducing batch effects while preserving cell type differences

First, to illustrate the contributions of scGCN for overcoming batch effects, we add comparisons with CCA on datasets of within-data, cross-platform, and cross-species scenarios that have true labels. Here we use both batch mixing entropy (Supplementary Fig. 4a) and cell-type mixing entropy (Supplementary Fig. 4b) as the evaluation indexes. Specifically, the batch mixing entropy shows the mixing level of cells from reference and query data[1]. A higher batch mixing entropy indicates better intermingling of cells from different data batches, wherein the reference and query data are regarded as two batches. The cell-type mixing entropy quantifies the intermingling of cells from different cell types[1]. A lower cell-type mixing entropy represents better separation of different cell types. Therefore, using these two evaluation indexes, we can measure the contributions of CCA and scGCN for eliminating batch effects while preserving the differences between different cell types.

Supplementary Fig. 4a shows the batch mixing entropy of scGCN and CCA respectively based on each paired reference-query dataset. Specifically, for within-data scenario that has no batch effects, CCA and scGCN both show higher batch mixing entropy. When applying to the reference-query pairs from different platforms and species, CCA and scGCN both show lower mixing entropy, indicating the underlying batch effects. Across all datasets, the batch mixing entropy of CCA does not show significant differences with that of scGCN (P value = 0.374). It suggests that scGCN has comparable capability with CCA in reducing batch effects from different datasets. Supplementary Fig. 4b shows the cell-type mixing entropy of scGCN and CCA respectively. We find that scGCN shows much lower cell-type mixing entropy than CCA, demonstrating that scGCN achieves better cell type separation. Across all datasets, the cell-type mixing entropy of scGCN is significantly lower than CCA (P value = 3.632e-12). It suggests that scGCN preserves cell type differences when applying to different datasets. Overall, these results show that CCA only mixes cells from different data batches but cannot retain cell type differences. In contrast, scGCN not only reduces batch effects but also avoids mixing cells from different cell types, demonstrating the contributions of our scGCN method in overcoming batch effects.

2). Evaluating ability of overcoming batch effects by label transfer performance

As mentioned by the reviewer, in the graph construction step of scGCN, canonical correlation analysis (CCA) is used to obtain batch-corrected low-dimensional space where inter-dataset mutual nearest neighbors (MNN) are identified. Here we use the CCA-MNN to identify the transferred labels, and compare with scGCN in their abilities of overcoming batch effects.

For CCA-MNN, we identify the transferred labels based on its graph. That is, for each cell in query data, its label is determined by its linked cells in reference data, according to the CCA-MNN graph.

In this way, the ability of CCA-MNN in overcoming batch effects for label transfer can be measured by the accuracy of identified cell labels in the query data. In Supplementary Fig. 5, we show the specific contributions of CCA-MNN and scGCN, using 26 datasets from three scenarios that have true labels, *i.e.*, within-data (Supplementary Fig. 5a), cross-platform (Supplementary Fig. 5b), and cross-species (Supplementary Fig. 5c). For each paired data, blue colored bar indicates the accuracy of CCA-MNN, and yellow bar indicates the increased accuracy of scGCN than CCA-MNN.

Supplementary Fig. 5a shows the accuracy of CCA-MNN and scGCN based on the 10 scRNA-seq datasets from within-data scenario (same data used in Fig. 2), whereby 50% of cells in each dataset are randomly selected as the reference data and the other 50% of cells as the query data. We find that across the 10 datasets, scGCN exceeds CCA-MNN with an average of 24.45% in accuracy, showing that the semi-supervised GCN component brings significant increase of accuracy, with the most increases observed in three datasets: SRP073767 with 53.36% increase, GSM3271044 with 44.96% increase, and GSM3271045 with 36.95% increase.

Supplementary Fig. 5b shows the accuracy of CCA-MNN and scGCN using the 12 reference-query pairs from the cross-platform datasets (same datasets with Fig. 3). We find that the semi-supervised GCN component of scGCN brings an average 60.83% increase of accuracy than CCA-MNN, with much more improvement than the within-data scenario (Supplementary Fig. 5a). CCA-MNN shows the best performance on three paired datasets, including GSE84133 – GSE85241 with 74.48% accuracy, GSE84133 – E-MTAB-5061 with 74.15% accuracy, and MCA 10x – MCA Smart-Seq2 with 61.67% accuracy, which are consistent with their less batch effects shown in Supplementary Fig. 4.

Supplementary Fig. 5c shows the accuracy of CCA-MNN and scGCN using the 4 reference-query pairs from different species (same datasets with Fig. 4). The yellow bar shows the increased accuracy contributed by the semi-supervised GCN component in scGCN. Similarly, CCA-MNN doesn't show decent accuracy in aligning datasets with substantial batch effects, with an average of 65.50% less accuracy than scGCN. The best accuracy of CCA-MNN is 50.99% when transferring labels across reference-query pair "GSE84133: human – mouse".

Overall, from the above comparisons, we find that CCA-MNN is not sufficient to overcome batch effects. The above results demonstrate the contributions of semi-supervised GCN model in overcoming batch effects and label transfer.

Note 2: Performance evaluation of scGCN using additional benchmarking data

To demonstrate that the superior performance of scGCN is independent to the selection of the reference datasets, we systematically examine scGCN in two aspects: 1). Performance comparisons are independent of reference data selection (Supplementary Fig. 6); 2). Systematic evaluation using benchmarking data (Supplementary Fig. 7). As Seurat v3 performs better than Conos, scmap, and CHETAH, we only show the comparisons between scGCN and Seurat v3.

1). Performance comparisons are independent of reference data selection

Here we examine whether the outperformance of scGCN depends on the selection of reference data in the reference-query data pairs. For each reference-query pair shown in our manuscript, we

reverse the pair and evaluate the performance. That is, the data previously used as reference is used as query, and vice versa. We do not evaluate the within-data scenario since the reference and query data are already randomly selected.

For the cross-platform (Fig. 3) and cross-species (Fig. 4) scenarios, there are a total of 11 unique combinations of reference and query data. The performance of these unique combinations is shown in Supplementary Fig. 6a, and the results of reversed pairs are shown in Supplementary Fig. 6b. In Supplementary Fig. 6a, as shown in our manuscript, scGCN has better performance with average accuracy of 86.6% than Seurat v3 (mean Acc= 78.6%). For these reversed pairs (Supplementary Fig. 6b), the average accuracy of scGCN is 86.9% that still outperforms Seurat v3 (mean Acc= 82.1%). These comparisons show the superior performance of scGCN is independent of our reference data selection.

2). Systematic evaluation using benchmarking data

We also agree with the reviewer that our selected datasets may not be representative. Therefore, we use a well-recognized benchmarking collection of single-cell datasets covering 13 major platforms[2] and completely examine all possible reference-query combinations, which allows unbiased evaluation of model performance.

As shown in Supplementary Fig. 7, we randomly pick one dataset from the 13 platforms as the reference and the other one as the query data. In this way, we get 156 different combinations of reference and query datasets from these platforms. The accuracy scores of scGCN versus Seurat v3 on all these 156 pairs are shown accordingly. Across all the 156 pairs, scGCN shows an average accuracy at 76.27% while Seurat v3 achieves the average accuracy at 71.12%. Specifically, scGCN shows better accuracy than Seurat v3 on 121 paired datasets with an average 7.28% increase of accuracy. On the other 34 paired datasets, Seurat v3 shows slightly higher accuracy with an average 2.30% increase of accuracy. scGCN's top outperformed cases are ICELL8 – inDrop, inDrop – CEL-Seq2, and MARS-Seq – Smart-Seq2, with increased accuracy of 33.91%, 32.21%, and 31.73%, respectively.

Altogether, with the comprehensive comparisons on reversed reference-query pairs and a collection of benchmarking data, scGCN consistently outperforms Seurat v3, which is not due to manually picked datasets.

Note 3: Computational scalability of scGCN on large-size single-cell data

We demonstrate the scalability of the scGCN method in large-scale datasets. Specifically, the memory usage and the computing time are profiled with respect to the sample size, from 100k up to 1 million cells, using the dataset generated by Cao J, et. al.[3]. The computational costs of scGCN are compared with Seurat v3 on a computer with 64 GB memory and 3.6 GHz Intel Core i9 processor. To enable label transfer for million-level or larger datasets, a batched approach is used for both methods. Briefly, the original dataset is split into batches for computing and the outputs from each batch are summarized into the final results. We show the computational time and average memory usage for different sample sizes (*i.e.*, 100k, 200k, …, 1millon) in Supplementary Fig. 8.

As shown in Supplementary Fig. 8, scGCN and Seurat v3 are comparable in computational time and memory usage. Specifically, in Supplementary Fig. 8a, the computing time of scGCN is lineally scalable with respect to the increase of sample size, ranging from 0.88 hours for 100k cells to 9.08 hours for 1 million cells, which are of the same magnitude with Seurat v3 that ranges from 0.48 hours to 5.11 hours respectively. Besides, we also investigate the batch-wise average memory usage of scGCN comparing with Seurat v3 (Supplementary Fig. 8b). scGCN shows lower memory usage (median: 2.11GB, IQR: 2.09 to 2.14GB) than Seurat v3 (median: 2.17GB, IQR: 2.14 to 2.19GB). Overall, scGCN is efficient and scalable for large-size single-cell dataset.

Regarding the batch-splitting strategy, we first construct reference and query datasets using Cao J, et. al.[3] that consists of 77 cell types and 4,062,980 cells, then split the query dataset into batches and independently learn cell labels for each batch, and finally summarize the results from each batch as the learned cell labels for the whole query dataset. 1) Data preparation. From Cao's original dataset, each cell type with more than 500 cells are retained for evaluation, thus resulting in 4,060,392 cells of 64 cell types. From these 4,060,392 cells, we randomly pick 200 cells for each of the 64 cell types and combine them as the reference data. From the rest of cells, we randomly sample cells as the query data. 2) Batch-splitting. The query data are then randomly split into batches, with each batch consisting of 5,000 cells. For example, if 1 million cells are randomly selected as the query data, totally 200 batches will be constructed. 3) Batch-splitting learning. For each batch of the query data, we use both scGCN and Seurat v3 to identify its transferred labels based on the reference data. 4) Summarization. The identified cell types for each batch are then summarized together to calculate the accuracy of the whole query data.

We next comprehensively investigate the accuracy of the batch-splitting approach comparing with the overall-learning approach for both scGCN and Seurat v3 at different query sample size scales, from 100k cells to 1 million cells. Here the "overall-learning" approach denotes using scGCN or Seurat v3 without batch-splitting. To present robust comparisons, for each query sample size, we repeat the whole experiment for 4 times, each with a new randomly sampled data. The label transfer accuracy is shown in Supplementary Fig. 9a. Different colors represent different strategies of scGCN and Seurat v3. scGCN's overall-learning (87.89%±0.23%) has slightly higher accuracy than its batch-splitting (85.93%±0.11%) across all query sample sizes. In contrast, for Seurat v3, the accuracy of the overall-learning approach (63.46% ± 3.48%) is much lower than the batch-splitting approach (76.85% ± 0.15%). In all cases, scGCN outperforms Seurat v3. Our analysis suggests that scGCN batch-splitting approach demonstrates comparable performance with scGCN overall-learning approach. Considering the superior scalability of the batch-splitting approach, in practice, we recommend using the batch-splitting approach when implementing scGCN on large query data.

To gain further insights into the scalability of the batch-splitting versus overall-learning, in Supplementary Fig. 9b, we apply both scGCN and Seurat v3 on query data ranging from 0.6k to 1 million cells using one of the four sampled datasets. As shown in the figure, scGCN and Seurat v3 demonstrate similar performance when cells are less than 2.5k, but they start to diverge on larger query datasets. Notably, when the query sample size is small (0.6k to 5k cells), the accuracies of both overall-learning and batch-splitting approaches of scGCN improve when the query sample size grows. When the query sample size is beyond 5k cells, both approaches reach maximum and stable performance. In contrast, though the Seurat v3's batch-splitting presents with small variations across different number of cells, the accuracy of the Seurat v3's overall-learning

approach gradually decreases when query data size is over 10k. This also explains why Seurat v3 shows lower accuracy of overall-learning in large-size dataset in Supplementary Fig. 9a.

The superior performance of scGCN over Seurat v3 on large query data may be due to that, as a semi-supervised approach, scGCN effectively learns the topological information from both reference and query data. A larger query data provides more topological information that benefits scGCN, and such benefit of a larger query data saturates at 5k cells. The performance of the Seurat v3 overall-learning approach deteriorates when the query data is over 10k cells, which might be due to that the large query data dominates the learning and dilutes the information from the reference data.

Overall, our results suggest that, in transfer-learning scenario, batch-splitting and overall-learning demonstrate similar accuracy when implementing scGCN on large query data. Therefore, scGCN is efficient and scalable for large-size single-cell dataset with high accuracy. We have revised the manuscript and cited Tran et.al[4] accordingly on Page 6 and in Supplementary Note 3. All detailed accuracy is deposited in the source data file.

Note 4: Effectiveness of the hybrid graph in scGCN

To verify that our graph construction is effective, we include different graph construction methods and compare them in two aspects: 1) Compare the accuracy of graphs constructed by CCA-MNN and other graph methods (Supplementary Fig. 10); 2) Evaluate the label transfer performance of GCN based on different graph construction methods (Supplementary Fig. 11). After these comparisons, we evaluate the robustness of GCN postprocessing (Supplementary Fig. 12).

Regarding the graph construction methods, we choose three alignment methods that have been proposed recently, including Scanorama[5], scmap-cell[6], and cellHarmony[7]. Scanorama first reduces dimensionality using randomized SVD, and then identifies mutual approximate nearest neighbors by locality sensitive hashing to construct the cell alignment graph. scmap-cell builds the alignment graph by performing the approximate nearest neighbor search using product quantization. This alignment graph is further pruned by cosine similarity between neighbored cells with the cutoff threshold of 0.5. cellHarmony first applies Louvain clustering on the k-nearest neighbor graph to identify community partitions, then identifies closest cells between two similar communities from reference and query data, thereby constructs the cell alignment graph.

Additionally, we further choose the kernel-based methods including tSNE[8], UMAP[9], and PHATE[10] as the other three graph construction methods. For tSNE, we construct the graph based on the similarity defined by symmetrized probability distribution using Gaussian kernel over cell pairs, in such a way that similar cells are assigned a higher probability while dissimilar cells are assigned a lower probability. For UMAP, we use the fuzzy graph, *i.e.,* the fuzzy simplicial set of the merged incompatible local views of the input data, which is pruned according to edge membership strength. For PHATE, we construct the graph using the potential distance information, which is computed as a divergence between the associated diffusion probability distributions of the two cells to all other cells, whereby the relationship of each cell to both near neighbors and distant points is accounted for in this distance.

The comparison results from our analyses are summarized below in three aspects.

1). Compare graph accuracy between CCA-MNN and other graph construction methods

To quantitatively evaluate different graph construction methods, we use the same 26 datasets with true labels in our manuscript to evaluate the performance in three scenarios, i.e., within-data, cross-platform, and cross-species, respectively.

First, for the within-data scenario, we use the same 10 scRNA-seq datasets of Fig. 2. For each dataset, 50% of its cells are randomly selected as the reference data and the other 50% of cells as the query data. Based on the reference-query data, the above six graph construction methods are used to learn both intra-data graph and inter-data graph. To evaluate different graph construction methods, we investigate the inter-graph accuracy and the intra-graph accuracy respectively (Supplementary Fig. 10a), which are defined as the proportions of correctly linked edges among all edges for inter-data graph and intra-data graph respectively.

As shown in Supplementary Fig. 10a, CCA-MNN consistently demonstrates superior performance across the 10 datasets, with both highest intra-graph accuracy (mean Acc = 93.4%) and inter-graph accuracy (mean Acc = 88.6%). Specifically, CCA-MNN is better than the alignment methods including Scanorama (intra: 82.6%, inter: 77.4%), scmap-cell (intra: 76.4%, inter: 72.9%), and cellHarmony (intra: 93.2%, inter: 73.9%). cellHarmony shows comparable accuracy with CCA-MNN on the intra-dataset graph, but not inter-dataset graph. Moreover, CCA-MNN also outperforms the kernel-based methods, *i.e.*, UMAP (intra: 68.2%, inter: 71.2%), tSNE (intra: 73.4%, inter: 75.3%), and PHATE (intra: 70.0%, inter: 73.6%). UMAP and PHATE show particularly lower accuracy relative to the other methods, especially in datasets GSE98638 and GSE99254. Interestingly, the overall accuracy pattern of all methods is aligned along the dashed line, indicating that intra-graph accuracy differs not much from inter-graph accuracy when there are no batch effects between reference and query data that are generated from the same dataset.

Second, we evaluate these graph construction methods using cross-platform datasets. Here we use the same 12 paired reference-query datasets as in Fig. 3. The reference and query data in each pair are profiled by different scRNA-seq technologies. We then apply the above methods to learn the intra-data graph and inter-data graph respectively. Similarly, we use both intra-graph accuracy and inter-graph accuracy to evaluate the performance of each graph construction method.

As shown in Supplementary Fig. 10b, CCA-MNN consistently demonstrates better performance than other methods across the 12 pairs of datasets, with both higher intra-graph accuracy (mean Acc = 97.9%) and inter-graph accuracy (mean Acc = 88.6%). Specifically, CCA-MNN is higher than the alignment methods including Scanorama (intra: 90.9%, inter: 76.4%), scmap-cell (intra: 90.2%, inter: 79.2%), and cellHarmony (intra: 96.9 %, inter: 69.5%). cellHarmony shows similar performance as CCA-MNN regarding the intra-dataset graph, but not the inter-dataset graph. Moreover, CCA-MNN also outperforms the kernel-based methods, *i.e.*, UMAP (intra: 83.3%, inter: 76.7%), tSNE (intra: 80.5%, inter: 67.8%), and PHATE (intra: 58.9%, inter: 37.1%). PHATE shows lower accuracy relative to other methods in certain reference-query pairs (*e.g.*, GSE81608 - GSE84133 and PBMC Seq-well - PBMC Smart-Seq2). Interestingly, the alignment methods tend to perform better than the kernel-based methods, except that UMAP shows comparable accuracy with the alignment methods. Different from the within-data scenario (Supplementary Fig. 10a), the overall accuracy pattern of all methods is shifting left from the dashed line, suggesting the substantial batch effects between reference and query data.

Lastly, we compare the graph construction methods using datasets from different species. Here we use the same four reference-query pairs of cross-species datasets as in Fig. 4. From Supplementary Fig. 10c, we find that all methods show comparably high intra-graph accuracy when leveraging human and mouse data from GSE84133, *i.e.*, CCA-MNN (intra: 99.5%, inter: 91.9%), Scanorama (intra: 97.9%, inter: 90.7%), scmap-cell (intra: 97.7%, inter: 77.6%), cellHarmony (intra: 98.0%, inter: 90.6%), UMAP (intra: 95.7%, inter: 79.1%), tSNE (intra: 93.3%, inter: 60.9%), and PHATE (intra: 97.1%, inter: 83.6%). However, CCA-MNN presents decent inter-graph accuracy (intra: 97.5%, inter:72.0%) when identifying graphs between phs001790 and GSE115746 datasets, whereas the other methods show much lower inter-graph accuracy, including Scanorama (intra: 94.9%, inter: 31.2%), scmap-cell (intra: 87.3%, inter: 38.5%), cellHarmony (intra: 98.5%, inter: 44.7%), UMAP (intra: 92.7%, inter: 21.9%), tSNE (intra: 92.8%, inter: 18.9%), and PHATE (intra: 97.2%, inter: 6%). Such lower inter-graph accuracy may be due to the substantial batch effects between phs001790 and GSE115746 datasets.

Altogether, the above results (Supplementary Fig. 10) show that CCA-MNN performs consistently better in identifying accurate hybrid graphs within data, cross platforms, and cross species.

2). Evaluate the label transfer performance of GCN based on different graph construction methods

After the comparisons of graph accuracy, we further evaluate the performance of GCN based on different graph construction methods. Here we use the same 26 datasets in our manuscript to evaluate the performance in three scenarios, *i.e.*, within-data, cross-platform, and cross-species, respectively.

First, we use the same 10 scRNA-seq datasets whereby 50% of cells in each dataset are randomly selected as the reference data and the other 50% of cells as the query data. We evaluate the performance of GCN based on different graph construction methods by the accuracy score (Acc), which is defined as the proportion of correctly predicted cells among all cells in the query data. As shown in Supplementary Fig. 11a, CCA-MNN+GCN (*i.e.*, scGCN) consistently demonstrates better performance with higher accuracy (mean Acc = 90.9%) than other methods across datasets. Specifically, CCA-MNN+GCN is higher than the alignment-based methods including Scanorama+GCN (86.0%), scmap-cell+GCN (84.3%), and cellHarmony+GCN (81.7%). CCA-MNN+GCN also outperforms the kernel-based methods, *i.e.*, UMAP+GCN (79.1%), tSNE+GCN (acc=80.5%), and PHATE+GCN (77.4%). Consistent with the results of graph accuracy in Supplementary Fig. 10a, UMAP and PHATE show relatively lower performance on datasets GSE98638 and GSE99254.

Second, we compare these methods on the reference-query datasets from different experimental platforms. Here we include the same 12 paired reference-query datasets used in Fig. 3. Each pair of reference-query datasets are profiled by different scRNA-seq technologies. Evaluated by the accuracy score, all methods present decent performance (Supplementary Fig. 11b), whereas CCA-MNN+GCN outperforms with consistently higher accuracy (mean Acc = 87.1%) than the alignment-based methods including Scanorama+GCN (82.9%), scmap-cell+GCN (85.9%), and cellHarmony+GCN (83.5%). These alignment-based methods achieve approximate performance with the kernel-based methods including UMAP+GCN (83.8%), tSNE+GCN (82.2%), but not

PHATE+GCN (76.2%). These results suggest that intra-graph, inter-graph, and GCN postprocessing all play important roles for accurate prediction.

Lastly, we compare these methods on the reference-query datasets from different species. Here we keep using the same four pairs of cross-species datasets as in Fig. 4. In Supplementary Fig. 11c, all methods show high performance when leveraging human and mouse data from GSE84133 (CCA-MNN+GCN: 95.5%; Scanorama+GCN: 91.6%; scmap-cell+GCN: 92.4%; cellHarmony+GCN: 92.3%; UMAP+GCN: 90.6%; tSNE+GCN: 93.3%; PHATE+GCN: 90.4%), which is consistent with their corresponding graph accuracy (Supplementary Fig. 10c). However, CCA-MNN+GCN presents adequate accuracy for predicting the labels of phs001790 or GSE115746 datasets, whereas the other methods show lower performance, which is also consistent with their inter-graph accuracy (Supplementary Fig. 10c).

3). Evaluate the robustness of GCN postprocessing

After comparing with the above graph construction methods, we further establish the robustness of the GCN postprocessing to the graph constructed by CCA-MNN. Here we include the total 26 paired reference-query datasets used as above, *i.e.*, within-data, cross-platform, and cross-species. For each reference-query pair, we first use CCA-MNN to construct hybrid graph and then repeatedly train GCN model using 10 different training/val/test set that are randomly split from reference data. Boxplots in Supplementary Fig. 12 show the stable accuracy of GCN based on the constructed graph in each pair of reference-query datasets. The average standard deviation of accuracy scores is 0.0017, 0.0038, and 0.0049 respectively in the three scenarios. Different colors represent different scenarios of paired datasets. This result confirms the robustness of GCN postprocessing to the CCA-MNN constructed graph.

In summary, through the comparisons of CCA-MNN with other graph construction methods based on both 1) the graph accuracy, 2) the label transfer performance, as well as 3) the robustness of GCN postprocessing based on CCA-MNN, our method shows superior and robust performance when transferring labels in within-data, cross-platform, and cross-species scenarios.

Note 5: Comparisons of GCN model with other graph neural network models

To validate the GCN model in scGCN, we choose the other six different graph neural networks for evaluation. They are Graph Attention Network (GAT)[11] that uses the multi-head self-attention mechanism to learn the hidden representations of graph-structured data for node classification; GraphSAGE[12] with LSTM and max-pooling aggregators that uses stochastic neighborhood sampling and aggregation for representation learning on large graphs; ChebyNet that convolutes graph-structured data using fast localized spectral filtering[13]; a most recently proposed method HyperGCN[14] that maps node features from Euclidean to hyperbolic space and implements hyperbolic graph convolution to learn inductive model for node representations; and another recent developed method, GWNN[15] that uses graph wavelet transform convolution operator rather than graph Fourier transform on graph data.

According to their published results, the above methods have been compared with GCN on semi-supervised classification of three benchmark datasets: Cora, Citeseer and Pubmed. Specifically, GAT outperforms GCN with 1.6% on Cora and Citeseer but not Pubmed dataset[11]. For GraphSAGE-LSTM and GraphSAGE-maxpool, we do not find their direct comparisons with GCN

in literature. ChebyNet shows respectively 0.3%, 0.5%, and 4.6% lower accuracy than GCN on Cora, Citeseer and Pubmed data[11]. HyperGCN is shown with 2% higher accuracy than GCN on Pubmed data but has 2% lower accuracy than GCN on Cora dataset[14]. HyperGCN does not show its performance on Citeseer dataset. GWNN is shown to outperform GCN with 1.3% on Cora and Citeseer datasets but not Pubmed dataset[15]. These reports show that, even though these methods use complicated formulation for graph convolution, their performance is marginally improved or even lower than GCN in these benchmarking datasets. Meanwhile, these graph neural network methods have not been evaluated for label transfer across different datasets.

To gain insights into their label transfer performance across different single-cell datasets, we compare these methods quantitatively. Here we use the same 26 datasets with true labels in our manuscript to evaluate their performance. That is, for comparisons in the within-data scenario, we use the same 10 scRNA-seq datasets from Fig. 2. For each dataset, 50% cells are randomly selected as the reference data and the other 50% cells as the query data. For comparisons in the cross-platform scenario, we use the same 12 paired reference-query datasets as in Fig. 3. Each pair of reference-query datasets are profiled using different scRNA-seq technologies. For comparisons in the cross-species scenario, we use the same four pairs of cross-species datasets from Fig. 4. The performance of each method is evaluated by the accuracy score (Acc).

The comparison results are summarized in Supplementary Fig. 13. Each panel shows the accuracy scores of scGCN versus one of the six methods on all 26 pairs of datasets. Different colors represent different paired reference-query datasets, while different symbols represent different label transfer scenarios, *i.e.*, within-data, cross-platform, cross-species.

Supplementary Fig. 13a shows the accuracy scores of each paired datasets for HyperGCN versus scGCN respectively. For HyperGCN, we use the 'HGCN' model with 'PoincareBall' manifold as it shows the best performance. The overall accuracy of HyperGCN does not show much difference with scGCN. The average difference of accuracy between HyperGCN and scGCN is 1.02%. The most different performances between HyperGCN and scGCN are shown on two datasets, *i.e.*, GSM3271044 and phs001790 (human) − GSE115746 (mouse). For the GSM3271044 dataset, HyperGCN outperforms scGCN with 3.15%. For the phs001790 (human) − GSE115746 (mouse) dataset, HyperGCN shows 5.58% less performance than scGCN. When transferring labels across all 26 datasets, HyperGCN outperforms scGCN in 10 datasets. The mean accuracy of HyperGCN on all datasets is 88.14%, which is slightly lower than scGCN (88.57%).

Supplementary Fig. 13b shows the accuracy scores of scGCN and GAT respectively based on each paired reference-query dataset. For GAT, we use 32 attention heads instead of the default 8 for better performance, and the same number of hidden units per each attention head in each layer as scGCN. The mean accuracy of GAT on all 26 datasets is 87.22%, which is a bit lower than scGCN (mean Acc = 88.57%). Among the 26 datasets, GAT slightly outperforms scGCN in 7 cases. Specifically, GAT shows better performance than scGCN on 6 of the 12 cross-platform datasets (increased accuracy of GAT over scGCN), *i.e.*, MCA 10x − MCA Smart-Seq2 (1.32%), E−MTAB−5061 − GSE84133 (0.28%), GSE81608 − GSE84133 (0.035%), PBMC DropSeq − PBMC10xV3 (1.09%), PBMC Cel−seq − PBMC InDrop (2.60%), PBMC InDrop − PBMC Cel−Seq (1.47%). Additionally, for the cross-species data, GAT is better than scGCN on datasets (GSE84133: mouse – human) with 0.7% increase of accuracy.

Supplementary Fig. 13c shows the accuracy scores of scGCN and GWNN respectively on each paired dataset. For GWNN, we use the same numbers of hidden units in each layer as scGCN. Interestingly, we find that the accuracy of GWNN is comparable with scGCN on each of the 26 datasets with minimal difference. The mean accuracy of GWNN on all 26 datasets is 87.28%, which is a bit lower than scGCN (mean Acc= 88.57%) but higher than GAT (mean Acc= 87.22%). Specifically, GWNN shows small increase of accuracy than scGCN on 6 of the 12 cross-platform datasets (increased accuracy of GWNN over scGCN), *i.e.*, MCA 10x − MCA Smart-Seq2 (0.767%), GSE85241 − GSE84133 (0.931%), E−MTAB−5061 − GSE84133 (0.103%), PBMC DropSeq − PBMC Smart-Seq2 (0.0869%), PBMCInDrop − PBMC Cel−Seq (0.549%). GWNN is shown underperform scGCN in other scenarios.

The other three methods show obviously lower accuracy than scGCN (Supplementary Fig. 13d-f). Supplementary Fig. 13d and Supplementary Fig. 13e show the accuracy scores of scGCN versus GraphSAGE-maxpool and GraphSAGE-LSTM respectively. We observe that scGCN has higher accuracy than both GraphSAGE-maxpool and GraphSAGE-LSTM models in almost all datasets, except that GraphSAGE-maxpool is 0.086% higher than scGCN in "PBMC DropSeq − PBMC Smart-Seq2", and GraphSAGE-LSTM is 1.79% higher than scGCN in GSM3271044 dataset. The mean accuracy of GraphSAGE-maxpool and GraphSAGE-LSTM on all 26 datasets is 77.50% and 77.7% respectively. Finally, for ChebyNet, the accuracy scores of scGCN versus ChebyNet are shown in Supplementary Fig. 13f. ChebyNet is applied with same parameters as scGCN. ChebyNet shows less accuracy in all datasets except the GSE72056 dataset, of which ChebyNet outperforms scGCN with 0.0193%. The mean accuracy of ChebyNet on all 26 datasets is 78.75 % that is lower than scGCN.

In the above comparisons, the six graph neural network methods do not show superior performance than scGCN. This may be due to 1) single-cell data with constructed graphs is different from their paper's benchmarking data (Cora, Citeseer, and Pubmed) where the graph originally exists; 2) the performance is evaluated for label transfer across two different datasets. Altogether, our comparisons demonstrate that graph neural network methods perform very well for single-cell label transfer, which also outperform current methods (Fig. 2-4). Among all network methods, scGCN shows best overall performance, meanwhile HyperGCN, GAT, and GWNN are also good alternatives. For user convenience, we also include the HyperGCN, GAT, and GWNN models in our scGCN tool to provide more options to users.

Note 6: Evaluate the capability of scGCN for detecting unknown cells

To show the capability of scGCN in detecting unknown cells, we provide comprehensive experiments to evaluate our method for identifying unknown cells in query data.

Statistical metrics

To identify potential unknown cells in query data, we provide a screening step in our scGCN model using two statistical metrics, entropy score and enrichment score, representing mixtureness and enrichment. Specifically, all cells in query data are grouped to different clusters by modularity-based community detection[16]. For each query cluster, we measure its mixtureness and enrichment based on the inter-data graph of scGCN. Our rationale is that, a query cluster of unknown cell type is more likely to have random links to different cell types in the reference data, while a query cluster of known cell type is more likely to link to a specific cell type in the reference data. In this

way, unknown cells can be identified by the two statistical metrics. Mathematical definitions are illustrated below.

Entropy score: For a cluster h in the query data, the mixtureness of this cluster is defined by the information entropy of normalized enrichment scores. That is,

$$H_h = -\sum_c^C \frac{S_{c,h}}{\sum_c^C S_{c,h}} \log \frac{S_{c,h}}{\sum_c^C S_{c,h}} \text{ and } S_{c,h} = \frac{m_{c,h}/\sum_c^C m_{c,h}}{n_c/\sum_c^C n_c}$$

, where c is a specific cell type, C is the set of all cell types in reference data, $m_{c,h}$ is the number of cells in query cluster h that are linked to cell type c in reference data by the inter-data graph of scGCN, and n_c is the number of cells belonging to cell type c in reference data.

Enrichment score: For a cluster h in the query data, the enrichment score ES_h is defined by the normalized enrichment of the most enriched cell type. That is,

$$ES_h = \max_{c \in C} S_{c,h} \Big/ \sum_c^C S_{c,h}$$

In summary, for a cluster in query data, the entropy score describes whether this cluster in query data is dominated by a specific cell type, and the enrichment score describes how strong this cell type is enriched. Thus, if the query cell cluster h has higher entropy and lower enrichment, these cells should be assigned as unknown cell types.

Performance evaluation

To demonstrate the performance of the two statistical metrics in identifying cells of unknown types, we perform a comprehensive evaluation experiment based on the cross-platform data and cross-species data used in Fig. 3 and Fig. 4. We construct evaluation datasets and evaluate the performance using the area under the receiver operating characteristics curves (AUC). Specifically, for a given reference-query data pair with l cell types in reference data, totally l evaluation datasets are derived, each with a specific cell type removed from the reference and thus the corresponding cells in query data are unknown. Both entropy score and enrichment score are calculated in each of the l evaluation datasets, and the overall performance of detecting unknown cells is summarized into AUC scores of the two metrices, respectively.

Based on the 12 reference-query pairs from different platforms and 4 pairs from different species, the performance of recognizing unknown cells is shown in the receiver operating characteristics (ROC) curves with calculated AUC values (Supplementary Fig. 14). For the paired datasets from different platforms, the ROC curves of entropy and enrichment scores are shown in Supplementary Fig. 14a. The average AUCs of entropy score and enrichment score are 0.857 and 0.814, respectively. Specifically, the entropy and enrichment scores both show high AUC for the GSE84133 – E-MTAB-5061 datasets. Similarly, for the paired datasets from different species, we identify the ROC curves of entropy and enrichment score in Supplementary Fig. 14b. The average AUCs of entropy score and enrichment score are 0.805 and 0.785, respectively. These two scores are shown with highest AUC for the GSE84133: human – mouse dataset. As the reference-query pair is from the same dataset that doesn’t have unknown cell types, we do not consider the within-

data scenario. Given the above evaluations, we demonstrate that the two statistical metrics in scGCN are effective in identifying unknown cells in query datasets.

Note 7: Compare scGCN with CCA-based Seurat v3 in label transfer

According to its vignette, Seurat v3 provides the options of using PCA as the default way (PCA-based Seurat v3) and CCA as an alternative (CCA-based Seurat v3) to project the structure of a reference onto the query, which are used in the anchor weighting and label transfer steps [17]. To investigate whether the CCA-based Seurat v3 gains better performance than PCA-based Seurat v3, we apply the CCA-based Seurat v3 to the 26 datasets from three scenarios that have true labels (Supplementary Fig. 15a). We also compare the performance of CCA-based Seurat v3 versus scGCN accordingly (Supplementary Fig. 15b). For evaluation, we use the accuracy score (Acc) to evaluate the performance of the CCA-based Seurat. In Supplementary Fig. 15, different colors represent different paired reference-query datasets, while different symbols represent different label transfer scenarios.

Supplementary Fig. 15a shows that PCA-based Seurat v3 has better performance on 20 of the total 26 datasets, except for 6 paired data including 1 reference-query pair from within-data, 2 from cross-platform, and 3 from cross-species scenario. Specifically, regarding the 4 reference-query data pairs from within-data (GSE118389), cross-platform datasets (MCA 10x − MCA Smart-Seq2, GSE84133 − GSE85241), and cross-species datasets (GSE84133: mouse – human), CCA-based Seurat shows limited increase of accuracy (0.0093%, 7.24%, 1.30%, and 3.16%). Interestingly, CCA-based Seurat shows higher accuracy (17.99% and 15.03% more than PCA-based Seurat) at the other two cross-species datasets, *i.e.*, phs001790 (human) − GSE115746 (mouse) and phs001790 (mouse) − GSE115746 (human). Across all 26 datasets, the mean accuracy of CCA-based Seurat is 78.96%, which is lower than PCA-based Seurat (mean Acc = 82.18%). These results indicate that PCA-based Seurat v3 performs better than CCA-based Seurat v3 in most cases. The observed better results of PCA-based Seurat v3 may because that it uses not only PCA in dimension reduction to identify individual sources of variation, but also CCA in constructing anchors to identify shared sources of variation across datasets. In contrast, CCA-based Seurat v3 uses redundant shared information that ignore individual variation of each dataset.

To further demonstrate our scGCN's performance, we compare it with CCA-based Seurat v3 in Supplementary Fig. 15b. We find that scGCN has better performance on all the 26 datasets. The mean accuracy of scGCN across all datasets is 88.57%, which is better than CCA-based Seurat v3 (mean Acc = 78.96%). Specifically, for the within-data "GSE108989", scGCN outperforms CCA-based Seurat v3 the most with 27.9% more accuracy. For the cross-platform dataset "MCA 10x − MCA Smart-Seq2", scGCN has 15.93% higher accuracy. Overall, these comparisons demonstrate the superior performance of scGCN than CCA-based Seurat v3.

References

1 Haghverdi, L., Lun, A. T. L., Morgan, M. D. & Marioni, J. C. Batch effects in single-cell RNA-sequencing data are corrected by matching mutual nearest neighbors. *Nature biotechnology* **36**, 421-427, doi:10.1038/nbt.4091 (2018).

2 Mereu, E. *et al.* Benchmarking single-cell RNA-sequencing protocols for cell atlas projects. *Nat Biotechnol* **38**, 747-755, doi:10.1038/s41587-020-0469-4 (2020).

3 Cao, J. *et al.* A human cell atlas of fetal gene expression. *Science* **370** (2020).

4 Tran, H. T. N. *et al.* A benchmark of batch-effect correction methods for single-cell RNA sequencing data. *Genome biology* **21**, 1-32 (2020).

5 Hie, B., Bryson, B. & Berger, B. Efficient integration of heterogeneous single-cell transcriptomes using Scanorama. *Nature Biotechnology* **37**, 685-691, doi:10.1038/s41587-019-0113-3 (2019).

6 Kiselev, V. Y., Yiu, A. & Hemberg, M. scmap: projection of single-cell RNA-seq data across data sets. *Nature Methods* **15**, 359-362, doi:10.1038/nmeth.4644 (2018).

7 DePasquale, E. A. K. *et al.* cellHarmony: cell-level matching and holistic comparison of single-cell transcriptomes. *Nucleic Acids Research* **47**, e138-e138, doi:10.1093/nar/gkz789 (2019).

8 Maaten, L. v. d. & Hinton, G. Visualizing data using t-SNE. *Journal of machine learning research* **9**, 2579-2605 (2008).

9 McInnes, L., Healy, J. & Melville, J. Umap: Uniform manifold approximation and projection for dimension reduction. *arXiv preprint arXiv:1802.03426* (2018).

10 Moon, K. R. *et al.* Visualizing structure and transitions in high-dimensional biological data. *Nat Biotechnol* **37**, 1482-1492, doi:10.1038/s41587-019-0336-3 (2019).

11 Veličković, P. *et al.* Graph attention networks. *arXiv preprint arXiv:1710.10903* (2017).

12 Hamilton, W., Ying, Z. & Leskovec, J. in *Advances in neural information processing systems.* 1024-1034.

13 Defferrard, M., Bresson, X. & Vandergheynst, P. Convolutional neural networks on graphs with fast localized spectral filtering. *Advances in neural information processing systems* **29**, 3844-3852 (2016).

14 Chami, I., Ying, Z., Ré, C. & Leskovec, J. in *Advances in neural information processing systems.* 4868-4879.

15 Xu, B., Shen, H., Cao, Q., Qiu, Y. & Cheng, X. Graph wavelet neural network. *arXiv preprint arXiv:1904.07785* (2019).

16 Waltman, L. & Van Eck, N. J. A smart local moving algorithm for large-scale modularity-based community detection. *The European physical journal B* **86**, 1-14 (2013).

17 Stuart, T. *et al.* Comprehensive integration of single-cell data. *Cell* **177**, 1888-1902. e1821 (2019).

Codes functionality

Here we use the example data to show the functionality of scGCN. In the example data, we include the data from Mouse (reference) and Human (query) of GSE84133 dataset. The reference dataset contains 1,841 cells and the query dataset contains more cells (N=7,264) and 12,182 genes, which can be used to reproduce the results of Fig. 4.

Details of scGCN

After download and install scGCN, we enter the scGCN folder and run the Rscript 'data_preprocess.R' to load and pre-process the example data by mainly using the function 'save_processed_data'. Then, we can run the 'train.py' script by default settings. All output will be shown in the 'output_log.txt' file, with the scGCN's performance shown at the bottom. Specifically, the arguments and parameters of two functions ('save_processed_data' and 'tran.py') are shown below.

Function

save_processed_data

Description

'save_processed_data' takes raw counts and labels of reference/query set to generate scGCN training input.

Usage

```
save_processed_data(
        count.list,
        label.list,
        Rgraph=TRUE,
        check_unknown=TRUE)
```

Arguments

count.list: list of reference data and query data; rows are genes and columns are cells
label.list: list of reference label and query label (if any), both are data frames with rownames identical with colnames of data; the first column is cell type
Rgraph: if Rgraph is TRUE, the hybrid graph will be generated by Rscript, we also provide alternative python generated hybrid graph that can be obtained by set Rgraph=FALSE.
check_unknown: if check_unknown=TRUE, the query data will be examined for potential unknown cells that belong to cell types not appearing in the reference set. If you use a comprehensive reference set, you may set check_unknown=FALSE.

Output

This function returns files saved in folders "input" & "process_data", that will be used in the 'train_py' file.

Examples

load count.list and label.list from folder "example_data"
save_processed_data(count.list,label.list, Rgraph=TRUE, check_unknown=FALSE)

Function
python train.py

Description
'train.py' uses the processed data for the scGCN training and obtain the accuracy of label transfer.

Usage
To run scGCN on your dataset, here we show an example:

```
import scGCN
python train.py --dataset=input --output=results --graph=True --model=scGCN
                 --learning_rate=0.01 --epochs=200 --hidden1=32  --dropout=0
                 --weight_decay=0 --early_stopping=10
```

Arguments
dataset: input folder of processed data
output: results folder with predicted results
graph: use the hybrid graph for scGCN training
model: use the GCN model for training
learning_rate: the initial learning rate during training process
epochs: the number of learning epochs
hidden1: the number of units in hidden layer
dropout: the dropout rate, i.e., 1 - keep probability
weight_decay: the weight for L2 loss on embedding matrix
early_stopping: the tolerance (the number of epochs) for early stopping

Output
The output files with scGCN predicted labels will be stored in the results folder.

Examples
python train.py --dataset=input
