# Supplementary material for: scGCN is a graph convolutional networks algorithm for knowledge transfer in single cell omics
Source: Nat Commun. 2021 Jun 22;12:3826. doi: 10.1038/s41467-021-24172-y (PMC8219725; doi:10.1038/s41467-021-24172-y)
Supplement: Supplementary file 3 — Reporting Summary [file 41467_2021_24172_MOESM3_ESM.pdf]

nature research

Corresponding author(s): Jing Su and Wei Zhang

Last updated by author(s): May 13, 2021

Reporting Summary

Nature Research wishes to improve the reproducibility of the work that we publish. This form provides structure for consistency and transparency in reporting. For further information on Nature Research policies, see our Editorial Policies and the Editorial Policy Checklist.

Statistics

For all statistical analyses, confirm that the following items are present in the figure legend, table legend, main text, or Methods section.

n/a	Confirmed	
☐	☒	The exact sample size (*n*) for each experimental group/condition, given as a discrete number and unit of measurement
☐	☒	A statement on whether measurements were taken from distinct samples or whether the same sample was measured repeatedly
☐	☒	The statistical test(s) used AND whether they are one- or two-sided<br>*Only common tests should be described solely by name; describe more complex techniques in the Methods section.*
☒	☐	A description of all covariates tested
☐	☒	A description of any assumptions or corrections, such as tests of normality and adjustment for multiple comparisons
☐	☒	A full description of the statistical parameters including central tendency (e.g. means) or other basic estimates (e.g. regression coefficient) AND variation (e.g. standard deviation) or associated estimates of uncertainty (e.g. confidence intervals)
☐	☒	For null hypothesis testing, the test statistic (e.g. *F*, *t*, *r*) with confidence intervals, effect sizes, degrees of freedom and *P* value noted<br>*Give P values as exact values whenever suitable.*
☒	☐	For Bayesian analysis, information on the choice of priors and Markov chain Monte Carlo settings
☒	☐	For hierarchical and complex designs, identification of the appropriate level for tests and full reporting of outcomes
☒	☐	Estimates of effect sizes (e.g. Cohen's *d*, Pearson's *r*), indicating how they were calculated

Our web collection on statistics for biologists contains articles on many of the points above.

Software and code

Policy information about availability of computer code

Data collection	No software was used.
Data analysis	All the functions mentioned above were implemented in the Python package scGCN, which are available at github (https://github.com/QSong-github/scGCN). Other tools and packages used in this paper include: Python version 3.7.5, numpy version1.19.5, tensorflow version 1.15.0, networkx version 2.2,pandas version 0.25.3, rpy2 version 3.2.2, scipy version 1.1.0, plotly version 4.3.0, umap-learn version 0.4.5, R version 3.6.1, CHETAH version 1.6.0, Seurat v3 version 3.2.3, scmap version 1.12.0, Conos version 1.4.0

For manuscripts utilizing custom algorithms or software that are central to the research but not yet described in published literature, software must be made available to editors and reviewers. We strongly encourage code deposition in a community repository (e.g. GitHub). See the Nature Research guidelines for submitting code & software for further information.

Data

Policy information about availability of data

All manuscripts must include a data availability statement. This statement should provide the following information, where applicable:

- Accession codes, unique identifiers, or web links for publicly available datasets
- A list of figures that have associated raw data
- A description of any restrictions on data availability

All datasets analyzed in the current study are publicly available that can be downloaded from their public accessions, including GSE108989 [https://www.ncbi.nlm.nih.gov/geo/query/acc.cgi?acc=GSE108989], GSE115746 [https://www.ncbi.nlm.nih.gov/geo/query/acc.cgi?acc=GSE115746], GSE118389 [https://www.ncbi.nlm.nih.gov/geo/query/acc.cgi?acc=GSE118389], GSE72056 [https://www.ncbi.nlm.nih.gov/geo/query/acc.cgi?acc=GSE72056], GSE98638 [https://www.ncbi.nlm.nih.gov/geo/query/acc.cgi?acc=GSE98638], GSE99254 [https://www.ncbi.nlm.nih.gov/geo/query/acc.cgi?acc=GSE99254], GSM3271044 [https://www.ncbi.nlm.nih.gov/geo/query/acc.cgi?acc=GSM3271044], GSM3271045 [https://www.ncbi.nlm.nih.gov/geo/query/acc.cgi?acc=GSM3271045], phs001790

[https://www.ncbi.nlm.nih.gov/projects/gap/cgi-bin/study.cgi?study_id=phs001790.v2.p1], SRP073767 [http://support.10xgenomics.com/single-cell/datasets], E-MTAB-5061 [https://www.ebi.ac.uk/arrayexpress/experiments/E-MTAB-5061], GSE84133 [https://www.ncbi.nlm.nih.gov/geo/query/acc.cgi?acc=GSE84133], GSE81608 [https://www.ncbi.nlm.nih.gov/geo/query/acc.cgi?acc=GSE81608], and GSE85241 [https://www.ncbi.nlm.nih.gov/geo/query/acc.cgi?acc=GSE85241]. The PBMC data of six different sequencing protocols are available from the Broad Institute Single Cell portal (https://portals.broadinstitute.org/single_cell/study/SCP424/single-cell-comparison-pbmc-data) and the Zenodo repository (https://doi.org/10.5281/zenodo.3357167). The multi-omics (scRNA-seq and scATAC-seq) data used in this study includes A549 data with accession number of GSM3271040 [https://www.ncbi.nlm.nih.gov/geo/query/acc.cgi?acc=GSM3271040] and GSM3271041 [https://www.ncbi.nlm.nih.gov/geo/query/acc.cgi?acc=GSM3271041], mouse brain data available in the 10x Genomics website (http://cf.10xgenomics.com/samples/cell-atac/1.1.0/atac_v1_adult_brain_fresh_5k/atac_v1_adult_brain_fresh_5k_filtered_peak_bc_matrix.h5) and Allen Institute website (http://celltypes.brain-map.org/api/v2/well_known_file_download/694413985), Kidney data with accession number of GSM3271045 [https://www.ncbi.nlm.nih.gov/geo/query/acc.cgi?acc=GSM3271045] and GSM3271044 [https://www.ncbi.nlm.nih.gov/geo/query/acc.cgi?acc=GSM3271044], and the Lung data from Tabula Muris [https://figshare.com/projects/Tabula_Muris_Transcriptomic_characterization_of_20_organs_and_tissues_from_Mus_musculus_at_single_cell_resolution/27733], GSE108097 [https://www.ncbi.nlm.nih.gov/geo/query/acc.cgi?acc=GSE108097], and GSE68103 [https://www.ncbi.nlm.nih.gov/geo/query/acc.cgi?acc=GSE68103].

Field-specific reporting

Please select the one below that is the best fit for your research. If you are not sure, read the appropriate sections before making your selection.

- [x] Life sciences
- [ ] Behavioural & social sciences
- [ ] Ecological, evolutionary & environmental sciences

For a reference copy of the document with all sections, see nature.com/documents/nr-reporting-summary-flat.pdf

Life sciences study design

All studies must disclose on these points even when the disclosure is negative.

Sample size	Not applicable.
Data exclusions	Not applicable.
Replication	Not applicable.
Randomization	Not applicable.
Blinding	Not applicable.

Reporting for specific materials, systems and methods

We require information from authors about some types of materials, experimental systems and methods used in many studies. Here, indicate whether each material, system or method listed is relevant to your study. If you are not sure if a list item applies to your research, read the appropriate section before selecting a response.

Materials & experimental systems

n/a	Involved in the study	
[x]	[ ]	Antibodies
[x]	[ ]	Eukaryotic cell lines
[x]	[ ]	Palaeontology and archaeology
[x]	[ ]	Animals and other organisms
[x]	[ ]	Human research participants
[x]	[ ]	Clinical data
[x]	[ ]	Dual use research of concern

Methods

n/a	Involved in the study	
[x]	[ ]	ChIP-seq
[x]	[ ]	Flow cytometry
[x]	[ ]	MRI-based neuroimaging
